# DNA repair and replication links to pluripotency and differentiation capacity of pig iPS cells

**DOI:** 10.1371/journal.pone.0173047

**Published:** 2017-03-02

**Authors:** Kai Liu, Jian Mao, Lipu Song, Anran Fan, Sheng Zhang, Jianyu Wang, Nana Fan, Na Liu, Xiaoying Ye, Haifeng Fu, Zhongcheng Zhou, Yong Wang, Hong Wei, Zhonghua Liu, Ziyi Li, Liangxue Lai, Xumin Wang, Lin Liu

**Affiliations:** 1 Experimental and Translational Research Center, Beijing Friendship Hospital, Capital Medical University, Beijing, China; 2 State Key Laboratory of Medicinal Chemical Biology, 2011 Collaborative Innovation Center for Biotherapy, College of Life Sciences, Nankai University, Tianjin, China; 3 Beijing Institute of Genomics, Chinese academy of Sciences, Beijing, China; 4 First Hospital, Jilin University, Changchun, China; 5 College of Life Science, Northeast Agricultural University, Harbin, China; 6 Guangzhou Institutes of Biomedicine and Health, Chinese Academy of Sciences, Guangzhou, China; 7 College of Basic Medical Sciences, Third Military Medical University, Chongqing, China; University of Texas at Austin Dell Medical School, UNITED STATES

## Abstract

Pigs are proposed to be suitable large animal models for test of the efficacy and safety of induced pluripotent stem cells (iPSCs) for stem cell therapy, but authentic pig ES/iPS cell lines with germline competence are rarely produced. The pathways or signaling underlying the defective competent pig iPSCs remain poorly understood. By improving induction conditions using various small chemicals, we generated pig iPSCs that exhibited high pluripotency and differentiation capacity that can contribute to chimeras. However, their potency was reduced with increasing passages by teratoma formation test, and correlated with declined expression levels of *Rex1*, an important marker for naïve state. By RNA-sequencing analysis, genes related to WNT signaling were upregulated and MAPK signaling and TGFβ pathways downregulated in pig iPSCs compared to fibroblasts, but they were abnormally expressed during passages. Notably, pathways involving in DNA repair and replication were upregulated at early passage, but downregulated in iPSCs during prolonged passage in cluster with fibroblasts. Our data suggests that reduced DNA repair and replication capacity links to the instability of pig iPSCs. Targeting these pathways may facilitate generation of truly pluripotent pig iPSCs, with implication in translational studies.

## Introduction

By ectopic expression of defined factors, Oct4, Sox2, Klf4 and c-Myc, somatic cells can be reprogrammed to induced pluripotent stem cells (iPSCs) [1, 2], providing an unlimited cell resource with potential for studying disease and use in regenerative medicine [3, 4]. iPSCs have been successfully generated from mouse and human, but it has been noted that mice may differ from humans in terms of pluripotency signaling and maintenance [5]. Pigs are similar to humans in anatomy, physiology and metabolism [6, 7] and also may provide a suitable source of xenotransplantation and a model for study of human diseases [8–11]. It is anticipated that derivation of pig iPSCs could complement with human research [12–15], including transplantation test in pre-clinical translational medicine, such as retinal [16] and myocardial therapy [17].

Pig iPSCs have been produced in many laboratories using various induction methods and show pluripotency to some degrees [12, 18–33]. piPSCs could pass the test of germ line chimera production at the molecular genotyping levels [22, 23], but stable chimerism remains to be determined (S1 Table). Thus far, pig iPSCs have not been shown to pass the crucial test of authentic pluripotency by generation of all-iPSC pigs through tetraploid embryo complementation. One obstacle is that exogenous genes in pig iPSCs could not be removed even under negative selection [34], suggesting that they are required to maintain self-renewal of pig iPSCs during passage [35]. Presumably, these might be related to incomplete epigenetic reprogramming including only limited telomere reprogramming [32], inferior culture conditions [26], or unique gene profile related to pig early embryo development and stem cell pluripotency [36, 37].

Induction efficiency and quality of iPSCs can be influenced by many factors. For instance, induction medium influences iPSC induction efficacy and quality. Knockout Serum Replacement (KSR) based medium facilitates generation of iPSCs, in contrast to original fetal bovine serum (FBS) based medium used for iPSC induction [38, 39]. Moreover, small molecules acting as epigenetic modifiers, e.g. BIX01294 (BIX, a G9a histone methyltransferase inhibitor) [40, 41], sodium butyrate (NaB, an histone deacetylase HDAC inhibitor) [42–45], or S-adenosylhomocysteine (SAH, a DNA demethylation agent) [46] enhances induction and quality of mouse and human iPSCs. Other small molecules such as 5-azacytidine (5-AZA, a DNA methyltransferase inhibitor), Valproic acid (VPA, another histone deacetylase inhibitors) also improve reprogramming and quality of iPSCs or porcine cloning efficiency by somatic cell nuclear transfer [47, 48].

Interestingly, we find that BIX or NaB facilitates telomere elongation in pluripotent stem cells by epigenetic modifications [49, 50]. We thought to test whether the small molecules can improve pig iPSC induction and quality using KSR-based medium by rigorous selection of large number of clones and by tests of embryoid body or teratoma formation and chimera production. Further, we performed RNA-sequencing experiments to understand the signalling pathways that are likely associated with pluripotency and differentiation capacity of pluripotent pig iPSCs.

## Materials and methods

### Generation of pig iPSCs

The care and use of mice and pigs for this research were based on the protocols of the animal research guidelines approved by the Institutional Animal Care and Use Committee (IACUC) of Nankai University, Jilin University, Northeast Agricultural University, and Guangzhou Institutes of Biomedicine and Health. The methods were carried out in accordance with the approved guidelines. The ethics committee specifically approved this study.

Taihu pig embryonic fibroblasts (PEF), Taihu adult pig fibroblasts (PF) or Small Xiang Pig PEFs with black coat were used for generation of pig iPSCs. Unless indicated, PEF from Small Xiang Pig were used in the experiments, and showed in all the figures except S4 Fig. Retroviruses were produced and harvested following the protocol described previously [32]. The day before plasmid transfection, 7×10^6^ 293-T cells were seeded per 100-mm dish. At 24h after the seeding of 293-T cells, pMXs-based retroviral vectors (pMXs-Oct4, Sox2, Klf4, c-Myc, and Nanog; these factors abbreviate to O, S, K, M, and N respectively), Gag-Pol and VSV-G (10:9:1) were introduced into 293-T cells using lipo-2000 transfection reagent according to the manufacturer’s instruction. Virus suspension was centrifuged at 80,000g at 4°C for 90min, the supernatants discarded and virus (packaged from one 100-mm dish) precipitation dissolved each with H-DMEM at 4°C overnight.

Prior to infection, 1 ×10^5^ pig embryonic fibroblast (PEF) or adult pig fibroblast (PF) at passage 3–5 were plated in a six-well dish. Cells were infected with pMXs-based retroviral vectors twice at 24-h interval, each for 12 h. After infection, the cells were induced under porcine iPSC medium containing Knock-out Dulbecco’s modified Eagle medium (KO-DMEM, Invitrogen), added with 20% knock-out serum replacement (KSR, Invitrogen), 100~500 Units/ml human leukemia inhibitory factor (hLIF, Millipore), 10ng/ml basic fibroblast growth factor (bFGF, Millipore), 0.1 mM β-mercaptoethanol (Sigma), 1 mM L-glutamine (Invitrogen), 0.1 mM nonessential amino acids (Sigma), and penicillin (100 U/mL)-streptomycin (100 μg/mL) (Invitrogen) and/or with NaB, SAH and BIX, depending on specific experiments. About 10 days after infection, 5×10^4^ cells were plated onto mitomycin C-inactivated MEF feeder cells, after which the medium was changed every day. ESC-like colonies were picked at days 14~20 following a standard protocol. Small molecules used in reprogramming or culture were purchased from Stemgent or Sigma and supplemented at the following final concentrations: 0.2 mM NaB, 0.4 μM SAH, 0.5 μM BIX, 1 μM PD0325901 (PD), 3μM CHIR99021 (CH), or 2 μM 5-azacytidine (AZA).

Small molecules or factors are provided also in Supplementary information and their concentration and combination listed as a table for testing the effect of bFGF and LIF in culture medium (S1 Fig), reprogramming factors OSKM, OSK and SKM (S2 Fig), and small molecules SAH, NaB, and BIX on iPSC induction (S3 Fig). In the end, we used combination of bFGF, hLIF, NaB, SAH, and BIX for iPSC induction (S4 Fig).

### Cell culture

Pig iPSCs were maintained on B6D2F1 mouse embryonic fibroblasts (MEFs) treated with mitomycin C in KO-DMEM medium supplemented with 20% Knock-out Serum Replacement (KSR, Invitrogen) or fetal bovine serum (FBS, Hyclone), penicillin (100 U/mL)-streptomycin (100 μg/mL) (Invitrogen), 0.1 mM nonessential amino acids (Sigma), 1 mM L-glutamine (Invitrogen), 0.1 mM β-mercaptoethanol (Sigma), 100 Units/mL hLIF (Millipore) and 10ng/ml bFGF (Millipore). The iPSCs were initially subcultured by mechanical method, and then passaged at a ratio of 1:4 every 4–6 days following digestion by 1 mg/mL collagenase IV (Sigma) or TrypLE (Invitrogen).

### Alkaline Phosphatase staining (AP)

AP activity was detected using an Alkaline Phosphatase Substrate Kit III (Vector, sk-5300), according to the manual instruction. AP-positive colonies under different conditions were counted and analysed by StatView software.

### Immunofluorescence microscopy

Immunofluorescence staining was performed as previously described [51]. Cells were washed in PBS, fixed in 3.7% paraformaldehyde, permeabilized with 0.1% Triton X-100, blocked with blocking solution, and incubated overnight at 4°C with primary antibodies Oct4 (1:200, sc9081, Santa Cruz), Nanog (1:200, ab80892, Abcam), Sox2 (1:200, ab5603, Millipore), SSEA-1 (1:200, MAB4301, Millipore), SSEA-4 (1:200, MAB4304, Millipore), H3K4me3 (1:200, ab1012, Abcam), H3K9ac (1:200, ab4441, Abcam), H3K27me3 (1:200, 07–449, Millipore), Dnmt3b (1:200, ab13604, Abcam), γH2AX (1:200, 05–636, Millipore) and Ki-67 (AB9260, Millipore). After wash with PBS for three times, cells were incubated with a secondary antibody [1:200, goat anti-mouse IgG (H+L) FITC (115-095-003; Jackson) and goat anti-rabbit IgG (H+L) AlexaFluor^®^ 594 (111-585-003; Jackson), or 488 goat anti-mouse IgM for SSEA1 (A2103, Invitrogen). Nuclei were stained in Vectashield medium (Vector) added with Hoechst 33342 (Sigma). Fluorescence images were captured using a Zeiss fluorescence microscope (AxioVision Z1). Antibody concentration and time of exposure were the same among different samples for semi-quantification. Specificity of antibodies was tested by peptide BLAST of NCBI, cellular localization, and staining between PEF and iPSCs. Fluorescence relative intensity was quantified using Image J software by subtraction of the background fluorescence.

### RNA extraction and quantitative real-time PCR (qPCR)

Total RNA was isolated from fibroblasts or iPSCs using RNeasy Mini Kit (Qiagen) and reverse transcribed using M-MLV Reverse Transcriptase (Invitrogen). cDNA was used as a template for qPCR. qPCRs were performed with the FastStart Universal SYBR Green Master (Roche) according to manufacturer’s instruction. Signals were detected using an iCycler iQ5 2.0 Standard Edition Optical System (Bio-Rad). Relative expression level of the target genes was normalized by β-actin. Primers were designed using the IDT DNA website or specified (S2 Table). All qPCRs were performed by more than three biological replicates, and the results indicated as means with error bars.

### Western blot

Cells were washed twice in PBS, collected, and lysed in sodium dodecyl sulfate (SDS) sample buffer on ice for 30 min and then sonicated for 1 min. After centrifugation at 10,000g and 4°C for 10 min, the supernatant was transferred into new tubes. The concentration of the protein sample was measured by bicinchoninic acid (Pierce BCA Protein Assay Kit, 23225). Twenty microgram total protein of each cell extract was resolved by 10% Bis-Tris Sodium dodecyl sulphate polyacrylamide gel electrophoresis and transferred to polyvinylidinedifluoride membrane (Millipore). Nonspecific binding was blocked by incubation in 5% non-fat milk or 5% BSA in Tris-buffered saline and Tween 20 at room temperature for 2 h. Blots were then probed overnight at 4°C with anti-β-tubulin (mouse monoclonal, AbM59005-37-PU), H3 (ab1791, Abcam), p53 (sc-126, Santa Cruz), H3K4me3 (ab1012, Abcam), H3K9ac (ab4441, Abcam), H3K9me3 (ab8898, Abcam), H3K27me3 (07–449, Millipore), γH2AX (05–636, Millipore). Immunoreactive bands were then probed for 2 h at room temperature with the appropriate horseradish peroxidase (HRP)-conjugated secondary anti-Rabbit IgG-HRP (GE Healthcare, NA934V) or goat anti-Mouse IgG (H+L)/HRP (ZSGB-BIO, ZB-2305). Protein bands were detected by Chemiluminescent HRP substrate (Millipore, WBKLS0500) and imaged using a Tanon western blot imager or by X-ray film exposure. H3 and β-tubulin served as loading control and for relative quantification of proteins tested.

### Fluorescence-Activated Cell Sorting (FACS) analysis

FACS analysis of porcine iPSCs was carried out using a BD LSR analyzer (BD Biosciences), and data analyzed by CELLQuest Pro. Antibodies used for this study were the same as those used for immunofluorescence indicated above. Immunostaining without primary antibodies served as negative controls.

### Four- to eight-cell mouse embryo injection

Approximately 5–10 pig iPSCs stained by DiI were injected into eight-cell mouse embryos as hosts using a Piezo injector as previously described [52].

### Teratoma formation and Haematoxylin and Eosin (HE) staining

Approximately 1×10^6^ iPSCs were subcutaneously injected into non-obese diabetic/severe combined immune deficient (NOD/SCID) mice. After one month or more, mice were sacrificed to assess teratoma formation. Teratomas were excised, fixed in 3.7% paraformaldehyde, washed in 70% ethanol, embedded in paraffin, and sectioned for histological examination by H&E staining.

### Differentiation *in vitro* by Embryoid Body (EB) formation test

Pig iPSCs were removed off feeder cells twice based on their differences in adherence to the bottom of dish. Then, cells were transferred to low-adhesive 35 mm non-coated plates and cultured in pig iPSC medium without bFGF and hLIF. Aggregated EBs were formed after 5–7 days and transferred onto gelatin-coated tissue culture dishes for differentiation for another 5–7 days. Differentiated cells were fixed for immunofluorescence staining using primary antibodies of three embryonic germ layers, including alpha 1-fetoprotein (AFP; DAK-N150130, DAKO) for endoderm, smooth muscle actin (SMA; ab5694, Abcam) for mesoderm, and β-III-tubulin (CBL412, Chemicon) for ectoderm. The secondary antibodies were the same as those for immunofluorescence staining as described above.

### Generation of chimeric pigs

*In vivo* ovulated and fertilized (IVO) by flushing, *in vitro* fertilized (IVF), or nuclear transfer (NT) pig embryos at 8-cell stage or blastocysts derived from female Yorkshire pigs were used as host embryos. Microinjection of pig iPSCs was performed in embryonic manipulation medium under oil at 39.8°C using Nikon inverted microscope equipped with micromanipulators. Pig iPSCs (20–30) with homogenous size in appearance were slowly injected into host embryos. Injected embryos were then cultured in PorcPRO E-Cleave medium (19982 = 3010; Minitube of America) until transfer into the recipients. About 12~85 embryos, depending on the sources of embryos (*in vivo*, IVF or NT embryos), were transferred into the distal tip of a uterine horn of each recipient female pig. During gestation, real-time ultrasonography examination was used to confirm and monitor pregnancy. Recipient pigs were separately maintained for normal gestating and farrowing sows. Piglets were born around 110 days and maintained for subsequent detection.

### RNA-sequencing and analysis

RNA from PEFs (at passage 5) and pig iPSCs (at passages 5 or 10) was extracted using a RNeasy Mini Kit (Qiagen, Cat#74104). Libraries for sequencing were generated using NEBNext^®^ Ultra ^™^ RNA Library Prep Kit for Illumina^®^ (NEB, Ipswich, MA) following manufacturer’s instruction, and index codes added to attribute sequences to each sample. The clustering of the index-coded samples was performed on a cBot Cluster Generation System using TruSeq PE Cluster Kit v3-cBot-HS (Illumia, Champaign, IL) according to the manufacturer’s instruction. After cluster generation, the library was sequenced on an Illumina Hiseq 2,000 platform and 100 bp paired-end reads were generated. Quality control of raw data (FASTQ format) was firstly processed through in-house Perl scripts. Clean data (clean reads) were obtained by removing reads with adapter and poly-N, as well as low abundant reads from raw data. Subsequent analyses were based on the clean data with high quality. Index of the reference genome was built using Bowtie v2.0.6 which is an ultrafast, memory-efficient short read aligner. Paired-end clean reads were aligned to the reference genome using TopHat v2.0.9 which is a fast splice junction mapper for RNA-Seq reads.

Multi-array log2 transformation, normalization, and t-test were performed to identify the differentially expressed genes between any two selected samples using genomic analysis software suite (http://www.geworkbench.org). Fragments per kilobase of exon per million fragments mapped (FPKM) were calculated for each gene. Hierarchical clustering was built using Pearson’s Correlation and total linkage algorithms. The FASTQ data described in this study have been uploaded to NCBI’s Gene Expression Omnibus (accession number: GSE87361). Gene Ontology (GO) analysis and Kyoto Encyclopedia of Genes and Genomes (KEGG) Pathway analysis of differentially expressed genes were performed using the Database for Annotation, Visualization and Integrated Discovery (DAVID); a hypergeometric test with the Benjamini and Hochberg false discovery rate (FDR) was performed using the default parameters to adjust the P value.

### Statistical analysis

All the experiments were performed more than three times (n≥3), and the mean ± standard error (SE) were shown. Statistical analysis of means and variance were compared by Fisher’s protected least-significant difference (PLSD) using StatView software from SAS Institute Inc (Cary, NC). Significant differences were defined as * p < 0.05, ** p < 0.01 and *** p<0.001.

## Results

### Induction of pig iPSCs by various factors or in combination with small molecules

It has been argued for sometime whether bFGF or LIF is required for pig iPSC induction, since bFGF is required for human iPSC/ESC derivation and culture, yet only LIF for mouse iPSC/ESC derivation and culture, and bFGF could harm mouse iPSC/ESC derivation. Firstly, we tested the induction of pig iPSCs by individual four factors (OSKM) or chemicals including bFGF, hLIF, PD, CH, NaB, BIX, and AZA, and evaluated the formation of iPSC colonies by AP positive staining. Only culture in KSR supplemented with bFGF or bFGF+hLIF generated iPSC colonies also shown by AP positive staining (S1 Fig). By various combinations, our data suggests that bFGF is essential and hLIF beneficial for pig iPSC derivation and culture. In the following experiments, KSR supplemented with bFGF and hLIF was used as basal iPSC induction medium.

**Fig 1:**
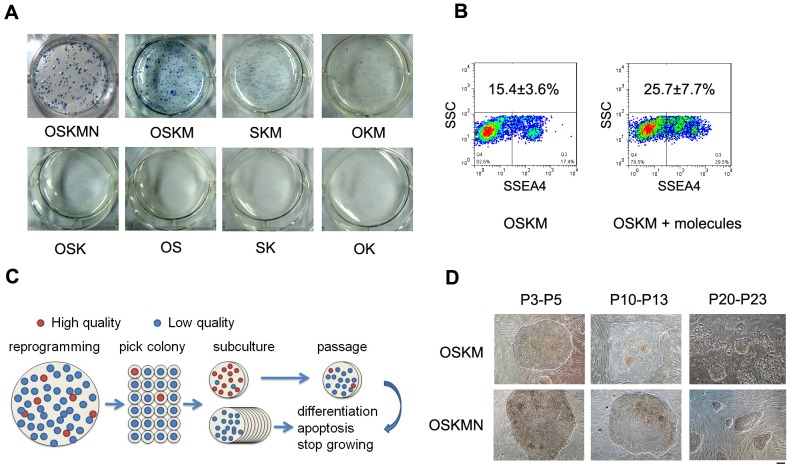
Generation of iPSCs from Pig Embryonic Fibroblasts (PEFs) using various factors and small molecules and by extensive clonal selections. **(A)** Colony identification by alkaline phosphatase (AP) positive staining during iPSC induction on day 18 by various combinations of the transcription factors, OCT4, O; SOX2, S; KLF4, K; c-MYC, M; NANOG, N. n = 3. **(B)** Small molecule compounds in combinations S-adenosylhomocysteine (SAH), sodium butyrate (NaB) and BIX01294 (BIX) improve the percentage of SSEA-4-positive cells by FACS analysis, n = 3. **(C)** Schematic outlining the strategy for colonies to be extensively picked and passaged to achieve high quality pig iPSCs. ESC-like colonies were picked at days 14~20 following a standard protocol. High-quality iPSCs were able to subculture by mechanical method, while low-quality iPSCs stoped growing or differentiated quickly before passage 5. However, High-quality iPSCs were difficult to maintain. (**D)** Phase contrast images showing representative pig iPSCs induced by OSKM or OSKMN at different passages. Scale bar = 100 μm.

A major problem for pig iPSCs is lack of silencing of exogenous genes following iPSC induction [35]. Then, we thought to employ fewer exogenous factors and tested their induction of pig iPSCs in KSR based media added with bFGF and hLIF but without small molecules. Two factors OS, SK, OK or three factors OSK failed to generate iPSC colonies by AP staining, and three factors OKM or SKM produced only few AP positive colonies. However, iPSCs induced by OKM or SKM were refractory to passage and exhibited limited AP positive staining in contrast to OSKM-iPSCs (Fig 1A and S2 Fig). These data show that four Yamanaka factors OSKM are required for induction of pig iPSCs from fibroblasts.

We also tested whether Nanog can enhance iPSC induction in combination with the four factors OSKM. OSKM produced large number of AP positive clones, and interestingly OSKMN produced relatively fewer AP positive clones but the clone size appeared larger relative to those of OSKM-induced iPSCs (Fig 1A). We chose OSKM and OSKMN as basic iPSC induction factors. Subsequently, pig iPSCs were induced and cultured in KSR medium supplemented with 100 Units/ml hLIF and 10 ng/ml bFGF.

Further, we tested whether small molecules could facilitate induction and quality of pig iPSCs, and assessed the iPSC clones by SSEA-4 expression by FACS. Following experiments for selection of small molecules, we found that small molecules (SAH, NaB, and BIX) nearly doubled SSEA-4 positive cells during reprogramming from day 1 to 18, compared to those clones formed in the controls without small molecules (Fig 1B and S3 Fig). These three small molecules can consistently facilitate generation of pig iPSCs, followed by culture in basic KSR medium added with 100 Units/ml hLIF and 10 ng/ml bFGF in the next experiments (S4 Fig).

Lastly, we combined bFGF, hLIF, SAH, NaB, and BIX to induce iPSCs and used KSR supplemented with bFGF and hLIF as basal culture medium. By extensively picking out a large number of colonies based on the strategy outlined in Fig 1C, we obtained several iPSC lines induced by OSKM or OSKMN (S4 Fig). These pig iPSCs resembled typical human ES cell colonies in morphology, flattened with clone boundaries but distinct from feeder fibroblasts (Fig 1D). The pig iPSCs exhibited large nuclei and visible nucleoli at higher magnification. The iPSCs thus generated by transcription factors during reprogramming with the small molecules were subsequently used in the functional and transcriptome analyses.

### Pluripotency *in vivo* of pig iPSCs

**Fig 2:**
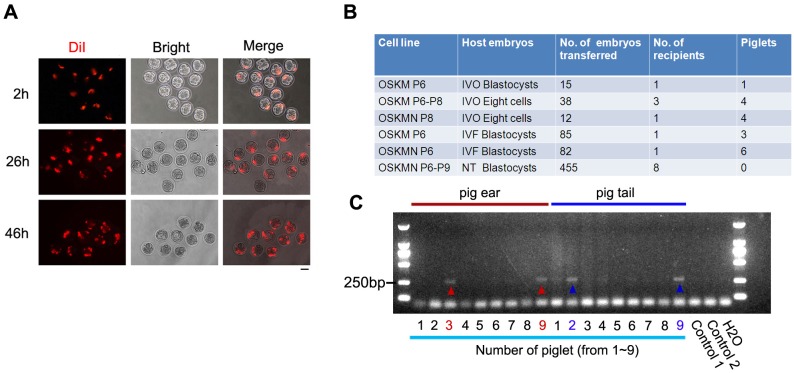
Pluripotency of pig iPSCs *in vivo*. **(A)** Bright-field optics and DiI fluorescence showing proliferation of pig iPSCs induced by OSKM at P10 following injection into 4-8-cell stage mouse embryos. Scale bar = 100 μm. **(B)** Summary table showing injection and chimera production of pig iPSCs induced by OSKM or OSKMN at different passages into pig embryos and their *in vivo* development. IVO, *in vivo* ovulated, and fertilization; IVF, *in vitro* fertilization; NT, nuclear transfer. **(C)** PCR analysis for the exogenous pig *Sox2* genes used in reprogramming of PEF to iPSCs showing that piglets’ ears (No. 3 and 9 piglets, indicated by red arrowheads) and piglets’ tails (No. 2 and 9 piglets, blue arrowheads) were positive for the exogenous gene. PEF (control 1/2) and water (H_2_O) served as negative controls.

Firstly, we investigated whether the newly established pig iPSCs are able to contribute to live pig offspring by chimera generation assay. We first employed mouse embryos to test the chimeric capacity of pig iPSCs. Pig iPSCs induced by OSKM at passage 10 labeled with a live fluorescent lipophilic cationic indocarbocyanine dye DiI were injected into a mouse embryo to examine whether they were able to incorporate to develop in mouse embryos. Five to ten cells labeled with red fluorescence were injected into a recipient 8-cell embryo, and one to two days after injection, 10–20 cells showed red fluorescence in the developed blastocyst (Fig 2A), suggestive of proliferation of iPSCs in the injected chimera embryo.

Next the pig iPSCs with black coat were injected into pig host embryos with white coat, obtained from *in vivo* ovulated and fertilized (IVO), *in vitro* fertilized (IVF), or nuclear transfer (NT). Live piglets were obtained and chimera formation was analyzed by coat color and genotyping (Fig 2B). A total of 687 chimera embryos were transplanted into 15 pseudo-pregnant pigs, 18 piglets generated, and all the 18 piglets were analyzed by PCR genotyping. These piglets showed no external abnormalities and no evident black coat of original donor pig cells. PCR analysis of genomic-integrated exogenous Sox2 was used to determine contribution of pig iPSCs in each offspring. PCR analysis of ear and tail biopsies indicated that ears of piglet No.3 (iPSCs induced by OSKM at passage 6–8) and No.9 (iPSCs induced by OSKMN at passage 8) and tails of piglet No.2 (iPSCs induced by OSKM at passage 6–8) and No.9 had incorporation of iPSCs into tissues (Fig 2C). These pig iPSCs produced three chimeras (16.7%) among 18 piglets by genotyping analysis. Other exogenous pluripotency genes were also tested, however we could not test them in tail or ear (data not shown).

### Characterization of pluripotency and differentiation capacity of pig iPSCs

**Fig 3:**
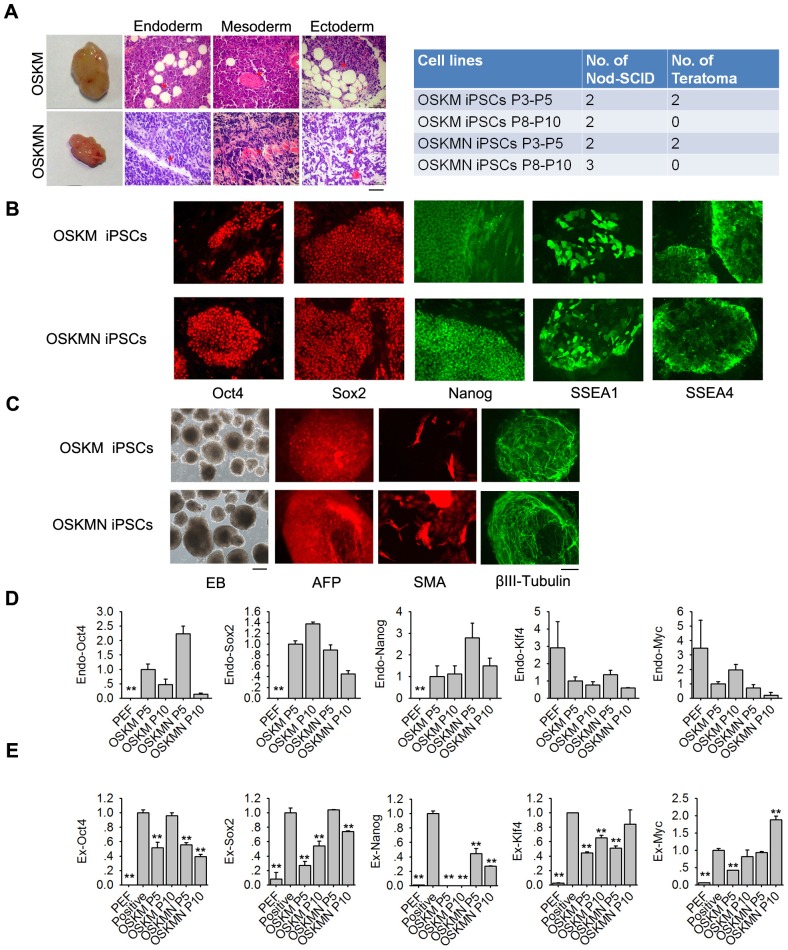
Characterization of pig iPSCs *in vitro*. **(A)** Teratoma formation test showing differentiation into epithelium (endoderm), muscle (mesoderm) and neural (ectoderm) by H&E histology of pig iPSCs induced from OSKM or OSKMN at passage 3–5. Scale bar = 50 μm. n = 4. **(B)** Expression of pluripotent markers, Oct4, Sox2, Nanog, SSEA1 or SSEA4 by immunofluorescence microscopy of OSKM or OSKMN-induced pig iPSCs at P10 (representative images were shown). n≥3. **(C)** Differentiation into three germ layers by embryoid body (EB) formation test of pig iPSCs induced by OSKM or OSKMN (representative images were shown). Endoderm marker, alpha 1-fetoprotein (AFP); mesoderm marker, smooth muscle actin (SMA); ectoderm marker, β-III-tubulin. For phase-contrast optics (Ph) of EB, scale bar = 100 μm, and for immunofluorescence images, scale bar = 50 μm. n≥3. **(D-E)** qPCR analysis for relative RNA expression of selected endogenous and exogenous pluripotency-associated genes in PEF, pig iPSCs induced by OSKM or OSKMN at passage 5 (P5) and passage 10 (P10). PEF without induction served as negative control and PEF induced by OSKMN on day 2 as positive control of exogenous genes. **, p<0.01. n≥3.

Next, we attempted to test the developmental potential *in vivo* of pig iPSCs using teratoma formation test by injection of the cells into immuno-deficient NOD/SCID mice. Notably, OSKM or OSKMN-induced iPSCs at passage 3–5 effectively formed teratomas within 4–8 weeks, consisting of representative derivatives of three germ layers as epidermis (ectoderm), muscle (mesoderm), and gland epithelium (endoderm) (n = 4). Rapid generation of teratomas can be indicative of the high differentiation capacity and thus high quality of iPSCs. Yet surprisingly the same iPSCs by passages 8–10 failed to produce teratomas within the same period of 4–8 weeks (n = 5) (Fig 3A). We did not test teratoma formation for longer time here, as we originally found that teratomas were formed by three months following injection of pig iPSCs [53].

Pig iPSCs by passage 10, regardless of induction by OSKM or OSKMN, still expressed multiple pluripotent stem cell markers as shown by immunofluorescence, including Oct4, Sox2 and Nanog in the nuclei, and SSEA-1 and SSEA-4 on cell surface (Fig 3B). Moreover, we assessed the differentiation capacity of pig iPSCs into three germ layers *in vitro* by standard embryoid body (EB) formation assay. Differentiation of OSKM or OSKMN-induced iPSCs via EB formation yielded three embryonic germ layers as evidenced by specific immunofluorescence staining of AFP (liver, endoderm), SMA (cardiac muscle, mesoderm), and β-III-tubulin (neurons, ectoderm) (Fig 3C). These data show that pig iPSCs by passage 10, induced by either OSKM or OSKMN, express pluripotent gene markers and are competent in differentiation *in vitro* into three germ layer lineages.

The qPCR analysis of PEFs and iPSCs revealed that expression levels of endogenous *Oct4*, *Sox2* and *Nanog* in iPSC lines induced by OSKM or OSKMN were dramatically higher than those of PEF, and expression of endogenous *Klf4* and *c-Myc* in iPSCs showed slight reduction (Fig 3D). These data suggest that both iPSCs at P5 and P10 show high expression of endogenous pluripotent genes.

Transgenes from the integrated retroviral vectors exhibited various expression levels, but were still not completely silenced in the derived pig iPSCs. Exogenous expression of *Oct4* was reduced at early passage in OSKM-induced iPSCs, but increased again to levels similar to positive controls by passage 10 and interestingly exogenous *Oct4* levels were reduced at both passage 5 and passage 10 in OSKMN-induced iPSCs. Exogenous *Sox2* showed reduced expression in OSKM-induced iPSCs but maintained active expression in OSKMN-induced iPSCs. Exogenous *Nanog* was absent in OSKM-induced iPSCs, consistent with only four factors without Nanog in induction of pig iPSCs here, and showed less expression in OSKMN-induced iPSCs. Exogenous *Klf4* was reduced in OSKM or OSKMN-induced iPSCs at early passage, but exogenous *c-Myc* maintained high expression in both types of iPSCs (Fig 3E).

### Transcriptome by RNA sequencing reveals pathways associated with competency of pig iPSCs

**Fig 4:**
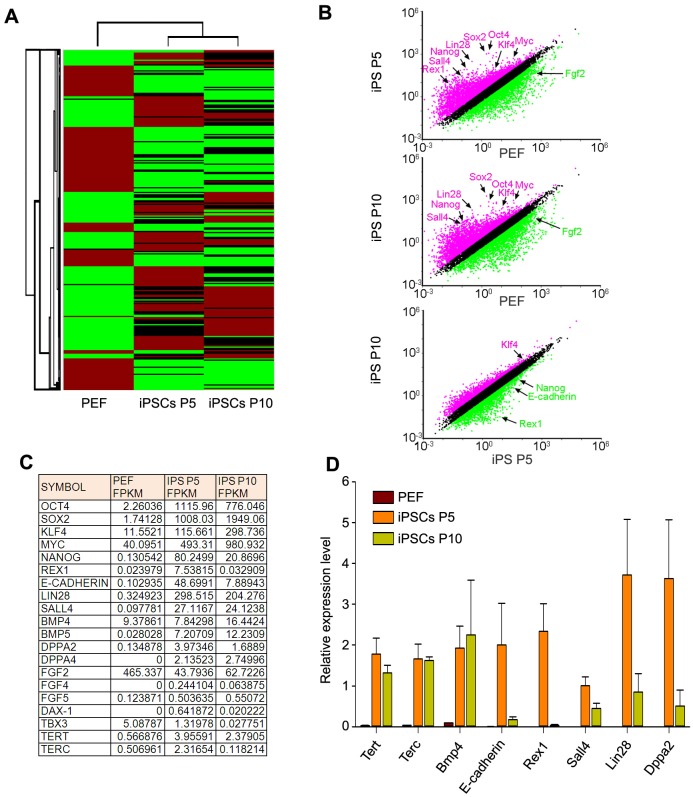
Hierarchical clustering and comparative analysis of pathways in pig iPSCs in comparison with PEFs by RNA-sequencing. **(A)** Hierarchical clustering and heat map of global gene expression patterns in PEF, pig iPSCs at P5, and at P10 by RNA-sequencing. All values are presented as mean of two independent experiments. **(B)** Scatter plots showing comparison of gene expression profile of PEF, pig iPSCs at P5 and pig iPSCs at P10. Both axes (in log10 scale) represent the gene expression values. Pink and green dots indicate genes with at least two-fold changes. **(C)** Comparative analysis of expression patterns of key pluripotency genes in PEF, and pig iPSCs at P5 and at P10. All values are presented as mean of two independent experiments. **(D)** qPCR analysis of expression of selected endogenous pluripotent genes in PEF and pig iPSCs at P5 and at P10. n≥3.

To understand potential molecular mechanisms underlying high pluripotency and differentiation competency of pig iPSCs at early few passages but rapid reduction in pluripotency and differentiation competency of pig iPSCs following additional passages, we performed RNA sequencing analysis of iPSCs at P5 which could contribute to chimeras, and iPSCs at P10 which had a slight differentiation, in comparison with progenitor PEF cells. We choose one cell line from OSKM and another cell line from OKSMN at passage 5 or 10 as parallel tests to improve test accuracy. Genome-wide transcriptome profiling indicates that pig iPSC lines (iPSCs P5 and iPSCs P10) clustered differently from progenitor somatic PEF cells (Fig 4A).

Comparative analysis of global gene expression pattern between iPSCs at P5 and PEF, iPSCs at P10 and PEF, iPSCs at P5 and P10 was conducted by scatter plotting (fold change >2, p <0.05). Pluripotency genes *Oct4*, *Sox2*, *Nanog*, *Klf4*, *Myc*, *Lin28*, and *Sall4* were highly upregulated in iPSCs at both P5 and P10, compared with PEF (Fig 4B). Total expression levels of *Klf4* or *c-Myc* with both endogenous and exogenous expression were higher in iPSCs than in PEF.

Interestingly, expression of pluripotency genes *E-cadherin*, *Rex1*, *Lin28*, and *Dppa2*, also naïve pluripotent markers for human and mouse ES/iPSCs [5, 54–56], were highly upregulated in iPSCs at P5 that retained developmental potential *in vivo*, but reduced in iPSCs at P10 that displayed reduced *in vivo* developmental potential (Fig 4C). Consistently, gene expression profile of iPSCs relative to PEF was validated by qPCR, showing that expression of *E-cadherin*, *Rex1*, *Lin28*, and *Dppa2* decreased significantly in pig iPSCs at later passage (Fig 4D).

We also analyzed signaling pathways involved in pig iPSCs at P5 and P10, in comparison with PEF. Briefly, *Tgfβ* was highly activated in PEF and downregulated after induction into iPSCs. In contrast, *Fst* an inhibitor of *Tgfβ* signaling was upregulated in iPSCs (S5 Fig). WNT signaling genes *Wnt8*, *Wnt11*.*1*, *Wnt11*.*2*, *Tcf*, and *Ruvbl (Ino80)* were upregulated in iPSCs at P5, but downregulated in iPSCs at P10 (S6 Fig). *Efg*, *Egfr*, *Pdgf*, and *Jnk* in MAPK pathway were highly expressed in PEF, but downregulated in iPSCs at early and late passage (S7 Fig). Moreover, signaling associated with apoptosis such as *Casp 3/8/9* was greatly reduced in iPSCs at P5 and P10. Some pro-apoptosis genes (e.g. *p53*) were increased in their expression, while anti-apoptosis genes (e.g. *Pik3*) upregulated in iPSCs (S8 Fig). Gene expression profile suggests that extensive reprogramming occurs during induction to pig iPSCs by addition of small molecules and following extensive clonal selections and that the signaling pathways support the high pluripotent state of pig iPSCs at early passages.

### DNA damage repair and replication pathways in pig iPSCs during passages

**Fig 5:**
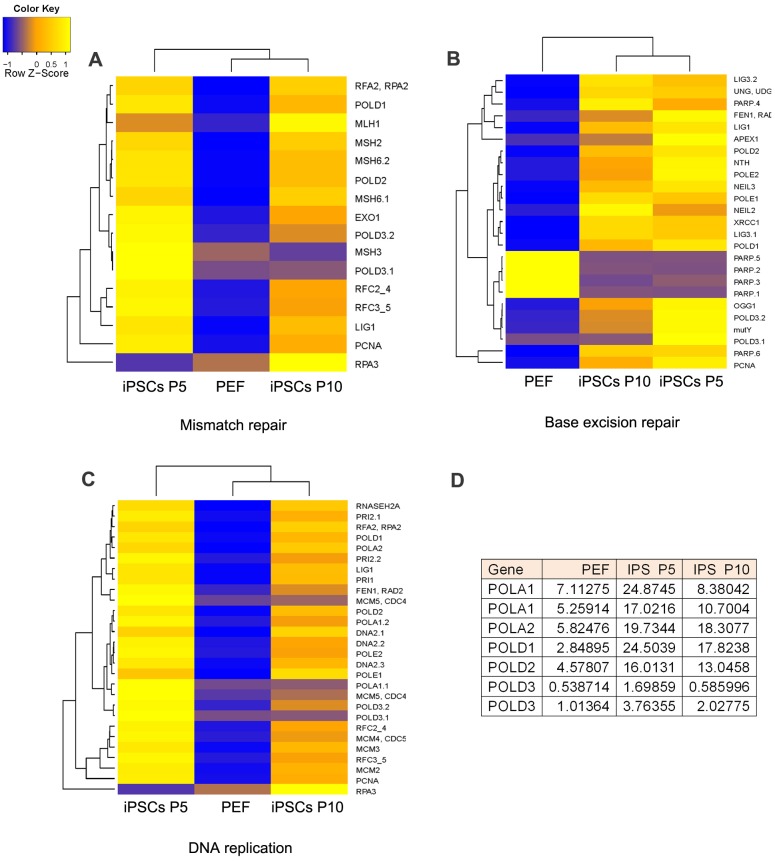
DNA repair and replication pathways of pig iPSCs revealed by RNA-sequencing and analysis. **(A-C)** DNA repair and replication pathways analyzed by RNA-sequencing, showing mismatch repair signal (A), base excision repair signal (B), and DNA replication signal (C). **(D)** List of selected genes associated with DNA repair and replication that exhibit higher expressions by RNA copy numbers in early passage pig iPSCs than those of PEFs but reduced expression following more passages. All values are presented as mean of two independent experiments.

Notably, pathways in DNA damage repair and DNA replication exhibited changes in pig iPSCs at P5 and P10, in comparison with PEF. Many genes for DNA repair and replication were upregulated in pig iPSCs at P5 but reduced by P10. Moreover, pig iPSCs at P10 were clustered with PEF in the expression profile of DNA repair and replication, in contrast to iPSCs at P5 (Fig 5A–5C).

DNA polymerase delta complex which involves in DNA replication and repair consists of 4 subunits: *Pold1*, *Pold2*, *Pold3*, and *Pold4*. In mismatch repair signaling pathway, *Pold1/2/3* expressed at lower levels in PEF and iPSCs at P10 than did iPSCs at P5. Consistently, signaling base excision repair (BER) was upregulated in iPSC at P5, but slightly reduced in iPSCs at P10 (Fig 5B). Nucleotide excision repair (NER) showed trends similar to BER (S9 Fig). Together, these data suggest reduced DNA replication and repair in pig iPSCs during prolonged passages.

**Fig 6:**
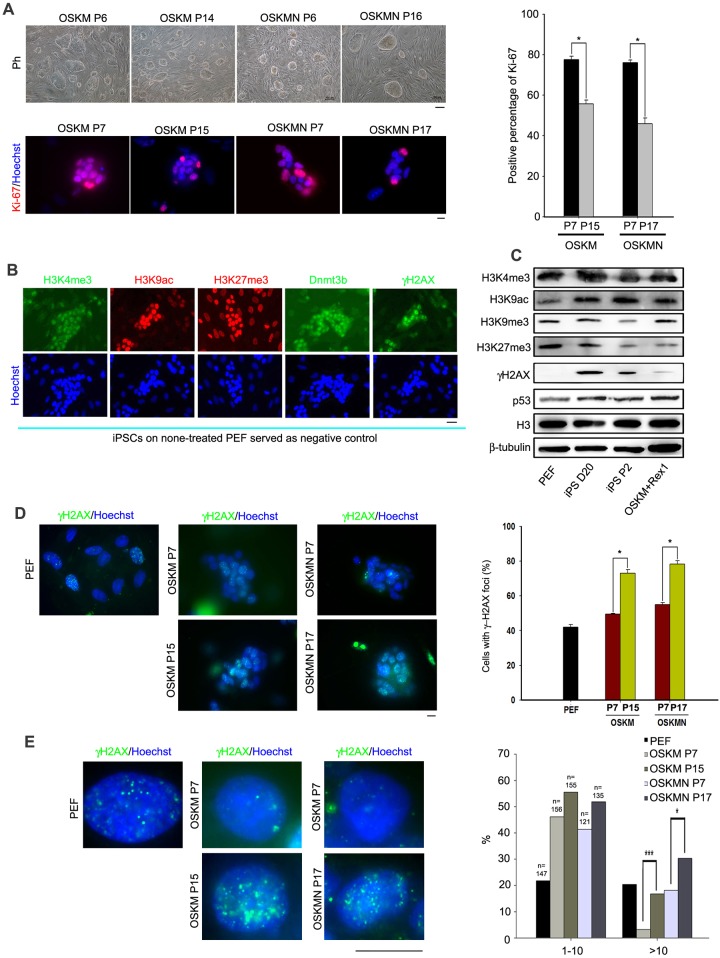
Cell proliferation, epigenetic modification and DNA damage response of pig iPSCs. **(A)** Reduced cell proliferation of pig iPSCs during prolonged passages. Left upper panel, phase contrast images of representative pig iPSCs and immunofluorescence staining of Ki-67 at early and late passages. Note, these iPSCs appeared as dome-shaped colonies, as they were cultured in the basal medium added with 5% ES quality FBS, so the cells proliferated faster, and could be passaged by single cell after digestion and separation using TrypLE. Right panel at bottom, percentage of Ki-67 positive cells in pig iPSCs induced by OSKM or OSKMN at early and late passages. *, p<0.05. For phase-contrast optics (Ph), scale bar = 100 μm; for immunofluorescence images, scale bar = 10 μm. **(B)** Epigenetic markers and DNA damage response of PEF and iPSCs at P2 by immunofluorescence staining. Nuclei were stained by Hoechst 33342 (lower panel). PEF at passage 3 as feeder layer served as background control by immunofluorescence. Scale bar = 20 μm. **(C)** Western blotting analysis of protein levels in PEF, iPSCs induced by OSKM on day 20, iPSCs induced by OSKM at P2 and iPSCs induced by OSKM+Rex1 at P2. H3 and β-tubulin served as loading control. **(D)** Left panel, representative images showing γH2AX immunofluorescence and foci in PEF and pig iPSCs at early and late passages induced by OSKM or OSKMN. Right panel, percentage of γH2AX-positve cells. *, p<0.05. Scale bar = 10 μm. **(E)** Left panel, images at higher magnification of γH2AX foci. Right panel, frequency of γH2AX foci in cells fewer than 10 and more than 10, respectively. *, p<0.05, ***, p<0.001. Scale bar = 10 μm. All the experiments, n≥3.

In addition, pig iPSCs at P10 clustered differently from those at P5 in DNA replication pathway (Fig 5C), implying that cell proliferation might also vary between early and late passages. Also considering, high expression levels of *Pold* and *Pola* in pig iPSCs at early passages but reduced levels at late passages (Fig 5D), we compared the cell proliferation rate between early and late passages of pig iPSCs by immunofluorescence of Ki-67. Percentage of cells with positive Ki-67 staining was greater in iPSCs at early passages (OSKM P7 or OSKMN P7) than at late passages (OSKM P15 or OSKMN P17) (Fig 6A), suggesting a declining proliferation with increasing passages of these pig iPSCs.

Further, we analyzed epigenetic reprogramming changes and DNA damage response in iPSCs at earlier passages using well recognized markers by immunofluorescence and western blot. Pig iPSCs at passage 2 expressed higher levels of H3K4me3, H3K9ac, H3K27me3, Dnmt3b, and γH2AX than did PEF (Fig 6B). By western blot, a decline in repressive histones (H3K9me3 and H3K27me3) and increase in active histones (H3K4me3 and H3K9ac) occurred during reprogramming (Fig 6C). However, protein levels of apoptosis-associated p53 in iPSCs were higher than those of PEF (Fig 6C), suggesting an increase in DNA damage response in pig iPSCs.

We assessed the DNA damage response by γH2AX immunofluorescence using the method described [57]. Both proportion of γH2AX positive cells (Fig 6D) and frequency of γH2AX foci (Fig 6E) increased with passages, indicating elevated DNA damage by passaging pig iPSCs. Consistently, pig iPSC clones exhibited signs of differentiation when propagated for more than 20 passages, such as changing to spindle morphology and lost clone morphology in Fig 1D at latest passage. Instability of pig iPSCs might be linked with increased DNA damage.

## Discussion

We have achieved pluripotent pig iPSCs induced in KSR-based medium supplemented with bFGF and hLIF in combination with small molecules that facilitate epigenetic reprogramming by intensive selection of clonal formation and we observed no significant differences between pig iPSCs induced by OSKM and OSKMN. These iPSCs at early passage exhibit high differentiation capacity by EB and teratoma formation tests, and are able to contribute to live chimeric offspring. However, their pluripotency and differentiation capacity decline rapidly following further passages. In our study, our pig iPS clones exhibited slight differentiation when propagated for more than 20 passages. We selected P3-5 and P8-10 to test developmental potential of our pig iPSCs. Therefore, it is possible that the pig iPSCs at passage >10 need longer time to form teratoma but we didn’t wait for such long time because we performed teratoma formation test of pig iPSCs at early and late passages at the same time. In our study, our pig iPSCs at passage 3–5 effectively formed teratomas within 4–8 weeks. In contrast, in study by Ezashi *et al*, pig iPSCs generated by them formed teratoma by three months after injection [18]. Similarly, in study by Esteban *et al*, their pig iPSCs also needed 9 weeks to form teratoma [20]. In study by Wu *et al*, they performed teratoma test used pig iPS lines at passage >10 and got teratoma by 4–6 weeks after injection. The large number of pig iPS cells they injected (5×10^6^) may contribute to the shorter time for the formation of teratoma. In spite of this, they used four pig iPS cell lines and three to five mice were injected for each cell line, but they only got three teratomas from two pig iPS cell lines [21]. Therefore, it is possible that pig iPSCs at early passages formed teratoma faster while at late passages needed longer time and this may be due to DNA damage during pig iPSCs culture. So the teratoma formation became difficult when pig iPSCs around passage 10 were injected into NOD/SCID mice.

Signalling pathway analysis indicates significant reprogramming in iPSC induction, including WNT, MAPK/ERK, TGFβ commonly implicated in iPSC/ESC self-renewal and pluripotency maintenance. Furthermore, we find that DNA damage repair and replication pathways are associated with reprogramming during pig iPSC induction, but they decline with increasing passages. Furthermore, TGFβ, WNT, MAPK, and apoptosis signalling are clustered in pig iPSCs at early and late passages but distinct from progenitor cells MEF. Specifically WNT signalling pathway is upregulated and MARK and TGFβ signalling pathway downregulated in pig iPSCs. Pig iPSCs generated by different methods and culture conditions may differ in their gene expression profile. Recently, most TGFβ and WNT pathways genes are not upregulated in pig iPSCs relative to mouse and human cells but BMP signalling pathway is dominant in pig iPSCs [36]. This is consistent with the increased BMP signalling in our pig iPSCs. On the other hand, aberrant silencing of imprinted genes, such as *Dlk1-Dio3* domain also may underlie the rare successful birth of chimeric offspring and limited ability to generate cloned animals from pig iPSCs [36]. However, use of KSR instead of FBS-containing medium is able to correct the aberrant imprinting [58, 59].

Genome stability is critical for maintaining pluripotency and differentiation capacity of iPSCs, and also for stem cell therapy in translational medicine. Telomere shortening and dysfunction can lead to genomic instability and aberrant karyotypes. Recently, we show that incomplete telomere reprogramming together with epigenetic reprogramming may contribute to instability of pig iPSCs [32]. Here we find by RNA-seq analysis that pathways in DNA damage repair and replication are reprogrammed during pig iPSC induction, and in early passage iPSCs, but reduced following additional passages, in association with decreased pluripotency and differentiation capacity.

DNA mismatch repair (MMR) is one of the several DNA repair pathways conserved from bacteria to humans to eliminate the mismatch of base-base insertions and deletions that appear as a consequence of DNA polymerase errors at DNA synthesis. Defects in genes encoding MMR enzymes (MMREs), MutS enzymes including Msh2, Msh3 and Msh6, and MutL enzymes including Mlh1, Mlh3, Pms1 and Pms2 can lead to cancer and neurodegenerative disease [60]. In addition, pig cells are prone to DNA and telomere damages [61]. Pig iPSCs at later passage exhibit reduced expression levels of *Msh3*, *Pold3*, and *Pold1*, and the DNA repair and replication pathway even cluster with progenitor PEF, in contrast to pig iPSCs at earlier passages.

Based on the assumption that pig iPSC colonies are not homogeneous, we screened iPSCs at passage 5 and continued passages by optimizing various induction and culture conditions and intensive selection of colonies, in an attempt to achieve homogeneous high-quality iPSCs and analyzed the iPSCs by RNA-sequencing. We were able to obtain several morphologically reasonably well pig iPSC lines that express typical pluripotency marker genes including those to mark naïve state. These pig iPSCs at early passages robustly show differentiation capacity to three germ layers by teratoma formation test. Our pig iPSCs also produced chimeric offspring by genotyping, but their germline competency needs to be tested in future experiments.

Pig iPSCs usually cannot maintain the pluripotency for a long time *in vitro*. There may be several reasons for this. First, the current medium for derivation and culture of pig iPSCs cannot enable the complete reprogramming of pig iPSCs. The most representative problem is the inadequate silence of exogenous genes in current pig iPSCs. Second, OSKM or OSKMN may be not the most suitable reprogramming factors combination for the induction of pig iPSCs. It is possible that pig iPSCs need specific factors other than Yamanaka factors. Third, in this study, we found that reduced DNA repair and replication capacity links to weakened pluripotency and differentiation capacity of pig iPSCs. Targeting on these three aspects may facilitate generation of authentic pig iPSCs, which can maintain pluripotency for long-term *in vitro*.

Pluripotent stem cells (PSCs) can be converted at two states: naïve and primed states, and only the naive PSCs can develop to chimeras when injected into allogeneic embryos while primed PSCs have very limited capacity to contribute to chimeric offspring [54]. Based on the expression of genes for naïve-like state, our pig iPSCs at early passage are more similar to naïve pluripotent state. Naïve iPSCs can be converted by various small molecule combinations, e.g. NHSM (LIF, TGFβ, bFGF, PD0325901, CHIR99021, SB203580 and SP600125) [5], 3iL (PD0325901, BIO, Dorsomorphin and LIF) [62], and 2i/L/b (PD0325901, CHIR99021, LIF and bFGF) [63]. Several naïve-like pig iPSCs have been reported [26, 27, 36]. Inhibition of MEK signaling combined with GSK3β inhibition and LIF supplementation were used to modulate pluripotency in porcine iPSCs. However, outcomes differ in different experimental settings. Under the stringent culture conditions together with small molecule inhibitors, pig iPSCs can acquire features of naive pluripotency, characterized with expression of *Stella* and *Rex1*, and increased *in vitro* germline differentiation capacity [64]. We also used 2i in the culure of pig iPSCs, but recently 2i showed a negative effect on pig iPSCs [65, 66]. By optimizing the culture condition that efficiently promotes mesenchymal-to-epithelial transition (MET), combination of three growth factors (LIF, FGF2 and BMP4) and two inhibitors (CHIR99021 and SB431542) could generate an intermediate pluripotent state of pig iPSCs, which were named as LFB2i-piPSCs [67]. Further optimization of induction and culture conditions by small molecules is still needed for achieving stable pig iPSCs that can maintain robust DNA repair and replication and high levels of *Rex1* with passages.

Together, during passaging of pig iPSCs, many genes for DNA repair and replication were reduced at late passage, which may be caused by the incomplete reprogramming of pig iPSCs. Accumulation of DNA damage and deficiency in DNA replication and repair likely underlie reduced pluripotency and differentiation capacity of pig iPSCs with advanced passages. Genomic integrity, DNA damage response, and failed apoptosis also are main concerns of human PSCs, and alleviation of the genomic insults and early detection of genomic instability will likely prevent the detrimental consequences of these genomic aberrations on PSC application in basic research and regenerative medicine [68]. We anticipate that targeting these specific pathways and factors would facilitate achieving stable truly pluripotent iPSCs for pre-clinical tests of efficacy and safety of stem cell therapy.

## Supporting information

S1 Fig bFGF and hLIF improve formation of AP positive iPSC colonies induced by OSKM. For the generation of high-quality pig iPSCs, a number of small molecules were tested. (A) Table summarizes induction factors, feeder, inducing medium, colony formation and AP staining. (B) Representative AP staining images under various conditions on day 12 of induction.
(DOC)

S2 Fig Pig iPSCs induced by OSKM are maintained better relative to fewer factors. iPSCs induced by OSKM exhibited strong AP staining in both KSR supplemented with bFGF and mTeSR, while OKM and SKM only showed no or weak AP staining at P3. Representative AP staining images (upper) and Table summary of colony picking and passaging of iPSCs (lower) are shown. Scale bar = 100 μm.
(DOC)

S3 Fig Addition of small molecules in the establishment of pig iPSCs. (A) Percentage of SSEA-4 positive cells was increased when small molecules in combination of NaB, SAH and BIX01294 (named 3 chemicals) were added from day 1 to day 18 during induction. (B) Table showing methods for selection and characteristics of pig iPSCs. (C) Representative images under bright field with phase contrast optics (left) and immunofluorescence staining and microscopy (right) of pig iPSCs induced by SKM or OSKM at P3. SKM-iPSC expressed Nanog and SSEA-4 but only weak Oct4 while OSKM-iPSC expressed Nanog, Oct4 and SSEA-4. Nuclei stained with Hoechst 33342 (blue). For phase-contrast optics (Ph), Scale bar = 100 μm; for immunofluorescence images, Scale bar = 50 μm.
(DOC)

S4 Fig High quality pig iPSCs induced by OSKM or OSKMN derived from Small Xiang Pig PEF by addition of small molecules. Taihu pig embryonic fibroblast (PEF), Taihu adult pig fibroblast (PF) and Small Xiang Pig PEF were used for generation of pig iPSC by OSKM or OSKMN. Through high throughput of picking colonies, the iPSCs were derived from Small Xiang Pig PEF. (A) Table summarizing characteristics of iPSC lines (upper) and representative images under bright-field with phase contrast optics under conditions numbered 1/3/4/5/6 (lower). Scale bar = 100 μm. (B) Expression levels of endogenous Nanog were significantly higher in colonies obtained underconditions No.5 and No.6 by qPCR. Representative high quality iPSCs colonies that could be cultured for more than 5 passages were examined.
(DOC)

S5 Fig TGF signaling pathway of pig iPSCs revealed by RNA-sequencing analysis. Tgfβ is highly activated in PEF and high expression of BMP in PEF is downregulated after induction into iPSCs. Interestingly, FST for inhibition of Tgfβ signaling is upregulated in iPSCs.
(DOC)

S6 Fig WNT signaling pathway of pig iPSCs revealed by RNA-sequencing. Wnt8, Wnt11.1, Wnt11.2, Tcf, and Ruvbl (Ino80) of Wnt signaling is upregulated in iPSCs at P5, but downregulated in iPSCs at P10.
(DOC)

S7 Fig MAPK signaling pathway revealed by RNA-sequencing. EFG, EGFR, PDGF, JNK of MAPK pathway are highly expressed in PEF, but downregulated in early and late passages of iPSCs in general.
(DOC)

S8 Fig Apoptosis signaling pathway analysis by RNA-sequencing. Signaling associated with apoptosis such as Casp3/8/9 is greatly reduced in iPSCs at P5 and P10. Some pro-apoptosis genes (p53) is upregulated, while anti-apoptosis genes (PIK3) also upregulated in iPSCs.
(DOC)

S9 Fig Nucleotide excision repair analysis by RNA-sequencing. Signaling associated with Nucleotide excision repair (NER) such as Pold1 and Pold3 is increased in iPSCs at P5 and P10.
(DOC)

S1 Table Characteristics of pig iPSCs produced by different laboratories. (DOC)

S2 Table Primers used for qPCR analysis. (DOC)

## References

[1] Takahashi K, Yamanaka S. Induction of pluripotent stem cells from mouse embryonic and adult fibroblast cultures by defined factors. Cell. 2006;126(4):663–76. Epub 2006/08/15. 10.1016/j.cell.2006.07.024 16904174

[2] Takahashi K, Tanabe K, Ohnuki M, Narita M, Ichisaka T, Tomoda K, et al Induction of pluripotent stem cells from adult human fibroblasts by defined factors. Cell. 2007;131(5):861–72. Epub 2007/11/24. 10.1016/j.cell.2007.11.019 18035408

[3] Yamanaka S. Induced Pluripotent Stem Cells: Past, Present, and Future. Cell Stem Cell. 2012;10(6):678–84. 10.1016/j.stem.2012.05.005 22704507

[4] Daley GQ. The Promise and Perils of Stem Cell Therapeutics. Cell Stem Cell. 2012;10(6):740–9. 10.1016/j.stem.2012.05.010 22704514 PMC3629702

[5] Gafni O, Weinberger L, Mansour AA, Manor YS, Chomsky E, Ben-Yosef D, et al Derivation of novel human ground state naive pluripotent stem cells. Nature. 2013;504(7479):282–6. 10.1038/nature12745 24172903

[6] Samstein B, Platt JL. Physiologic and immunologic hurdles to xenotransplantation. J Am Soc Nephrol. 2001;12(1):182–93. 11134266 10.1681/ASN.V121182

[7] VodiČKa P, Smetana K, DvoŘÁNkovÁ B, Emerick T, Xu YZ, Ourednik J, et al The Miniature Pig as an Animal Model in Biomedical Research. Ann N Y Acad Sci. 2005;1049(1):161–71. 15965115 10.1196/annals.1334.015

[8] Cozzi E, Bosio E, Seveso M, Rubello D, Ancona E, Jain S, et al Xenotransplantation as a model of integrated, multidisciplinary research. Organogenesis. 2009;5(1):288–96. 19568350 10.4161/org.7578 PMC2659370

[9] Giraud S, Favreau F, Chatauret N, Thuillier R, Maiga S, Hauet T. Contribution of Large Pig for Renal Ischemia-Reperfusion and Transplantation Studies: The Preclinical Model. J Biomed Biotechnol. 2011;2011:532127 10.1155/2011/532127 21403881 PMC3051176

[10] Whyte JJ, Prather RS. Genetic Modifications of Pigs for Medicine and Agriculture. Mol Reprod Dev. 2011;78(10–11):879–91. 10.1002/mrd.21333 21671302 PMC3522184

[11] Adam SJ, Counter CM. A method to generate genetically defined tumors in pigs. Methods in enzymology. 2008;439:39–51. 10.1016/S0076-6879(07)00404-1 18374155 PMC2688827

[12] Montserrat N, Bahima EG, Batlle L, Hafner S, Rodrigues AM, Gonzalez F, et al Generation of pig iPS cells: a model for cell therapy. J Cardiovasc Transl Res. 2010;4(2):121–30. Epub 2010/11/20. 10.1007/s12265-010-9233-3 21088946

[13] West F, Stice S. Progress toward generating informative porcine biomedical models using induced pluripotent stem cells. Ann N Y Acad Sci. 2011;1245:21–3. Epub 2012/01/04. 10.1111/j.1749-6632.2011.06337.x 22211969

[14] Esteban MA, Peng M, Deli Z, Cai J, Yang J, Xu J, et al Porcine induced pluripotent stem cells may bridge the gap between mouse and human iPS. IUBMB life. 2010;62(4):277–82. 10.1002/iub.307 20101630

[15] Roberts RM, Telugu BP, Ezashi T. Induced pluripotent stem cells from swine (Sus scrofa): why they may prove to be important. Cell cycle. 2009;8(19):3078–81. 10.4161/cc.8.19.9589 19738434

[16] Zhou L, Wang W, Liu Y, Fernandez de Castro J, Ezashi T, Telugu BP, et al Differentiation of induced pluripotent stem cells of swine into rod photoreceptors and their integration into the retina. Stem cells. 2011;29(6):972–80. 10.1002/stem.637 21491544 PMC4263955

[17] Li X, Zhang F, Song G, Gu W, Chen M, Yang B, et al Intramyocardial Injection of Pig Pluripotent Stem Cells Improves Left Ventricular Function and Perfusion: A Study in a Porcine Model of Acute Myocardial Infarction. PloS one. 2013;8(6):e66688 10.1371/journal.pone.0066688 23805264 PMC3689724

[18] Ezashi T, Telugu BP, Alexenko AP, Sachdev S, Sinha S, Roberts RM. Derivation of induced pluripotent stem cells from pig somatic cells. Proc Natl Acad Sci U S A. 2009;106(27):10993–8. Epub 2009/06/23. 10.1073/pnas.0905284106 19541600 PMC2698893

[19] Telugu BP, Ezashi T, Roberts RM. Porcine induced pluripotent stem cells analogous to naive and primed embryonic stem cells of the mouse. Int J Dev Biol. 2011;54(11–12):1703–11. Epub 2011/02/10. 10.1387/ijdb.103200bt PMC9006238 21305472

[20] Esteban MA, Xu J, Yang J, Peng M, Qin D, Li W, et al Generation of induced pluripotent stem cell lines from Tibetan miniature pig. J Biol Chem. 2009;284(26):17634–40. Epub 2009/04/21. 10.1074/jbc.M109.008938 19376775 PMC2719402

[21] Wu Z, Chen J, Ren J, Bao L, Liao J, Cui C, et al Generation of pig induced pluripotent stem cells with a drug-inducible system. J Mol Cell Biol. 2009;1(1):46–54. Epub 2009/06/09. 10.1093/jmcb/mjp003 19502222

[22] West FD, Terlouw SL, Kwon DJ, Mumaw JL, Dhara SK, Hasneen K, et al Porcine induced pluripotent stem cells produce chimeric offspring. Stem Cells Dev. 2010;19(8):1211–20. Epub 2010/04/13. 10.1089/scd.2009.0458 20380514

[23] West FD, Uhl EW, Liu Y, Stowe H, Lu Y, Yu P, et al Brief report: chimeric pigs produced from induced pluripotent stem cells demonstrate germline transmission and no evidence of tumor formation in young pigs. Stem cells. 2011;29(10):1640–3. Epub 2011/11/01. 22039609 10.1002/stem.713

[24] Ruan W, Han J, Li P, Cao S, An Y, Lim B, et al A novel strategy to derive iPS cells from porcine fibroblasts. Sci China Life Sci. 2011;54(6):553–9. Epub 2011/06/28. 10.1007/s11427-011-4179-5 21706416

[25] Montserrat N, de Onate L, Garreta E, Gonzalez F, Adamo A, Eguizabal C, et al Generation of feeder-free pig induced pluripotent stem cells without Pou5f1. Cell Transplant. 2011;21(5):815–25. Epub 2011/09/29. 21944493 10.3727/096368911X601019

[26] Cheng D, Guo Y, Li Z, Liu Y, Gao X, Gao Y, et al Porcine induced pluripotent stem cells require LIF and maintain their developmental potential in early stage of embryos. PloS one. 2012;7(12):e51778 Epub 2012/12/20. 10.1371/journal.pone.0051778 23251622 PMC3522612

[27] Fujishiro SH, Nakano K, Mizukami Y, Azami T, Arai Y, Matsunari H, et al Generation of naive-like porcine-induced pluripotent stem cells capable of contributing to embryonic and fetal development. Stem Cells Dev. 2012;22(3):473–82. Epub 2012/08/15. 10.1089/scd.2012.0173 22889279 PMC3549629

[28] Kues WA, Herrmann D, Barg-Kues B, Haridoss S, Nowak-Imialek M, Buchholz T, et al Derivation and characterization of sleeping beauty transposon-mediated porcine induced pluripotent stem cells. Stem Cells Dev. 2012;22(1):124–35. Epub 2012/09/20. 10.1089/scd.2012.0382 22989381

[29] Thomson AJ, Pierart H, Meek S, Bogerman A, Sutherland L, Murray H, et al Reprogramming pig fetal fibroblasts reveals a functional LIF signaling pathway. Cell Reprogram. 2012;14(2):112–22. Epub 2012/02/22. 22339199 10.1089/cell.2011.0078

[30] Zhang Y, Wei C, Zhang P, Li X, Liu T, Pu Y, et al Efficient reprogramming of naive-like induced pluripotent stem cells from porcine adipose-derived stem cells with a feeder-independent and serum-free system. PloS one. 2014;9(1):e85089 Epub 2014/01/28. 10.1371/journal.pone.0085089 24465482 PMC3896366

[31] Kwon DJ, Jeon H, Oh KB, Ock SA, Im GS, Lee SS, et al Generation of leukemia inhibitory factor-dependent induced pluripotent stem cells from the Massachusetts General Hospital miniature pig. Biomed Res Int. 2013;2013:140639 Epub 2013/12/29. 10.1155/2013/140639 24371815 PMC3858863

[32] Ji G, Ruan W, Liu K, Wang F, Sakellariou D, Chen J, et al Telomere reprogramming and maintenance in porcine iPS cells. PloS one. 2013;8(9):e74202 Epub 2013/10/08. 10.1371/journal.pone.0074202 24098638 PMC3787036

[33] Fan N, Chen J, Shang Z, Dou H, Ji G, Zou Q, et al Piglets cloned from induced pluripotent stem cells. Cell Res. 2013;23(1):162–6. 10.1038/cr.2012.176 23247628 PMC3541650

[34] Du X, Feng T, Yu D, Wu Y, Zou H, Ma S, et al Barriers for Deriving Transgene-Free Pig iPS Cells with Episomal Vectors. Stem cells. 2015;33(11):3228–38. 10.1002/stem.2089 26138940 PMC5025037

[35] Hall VJ, Kristensen M, Rasmussen MA, Ujhelly O, Dinnyes A, Hyttel P. Temporal repression of endogenous pluripotency genes during reprogramming of porcine induced pluripotent stem cells. Cell Reprogram. 2012;14(3):204–16. Epub 2012/05/15. 22578162 10.1089/cell.2011.0089

[36] Cao S, Han J, Wu J, Li Q, Liu S, Zhang W, et al Specific gene-regulation networks during the pre-implantation development of the pig embryo as revealed by deep sequencing. BMC Genomics. 2014;15:4 Epub 2014/01/05. 10.1186/1471-2164-15-4 24383959 PMC3925986

[37] Liu S, Bou G, Sun R, Guo S, Xue B, Wei R, et al Sox2 is the Faithful Marker for Pluripotency in Pig: Evidence From Embryonic Studies. Dev Dyn. 2015;244:619–27. 10.1002/dvdy.24248 25619399

[38] Zhao XY, Li W, Lv Z, Liu L, Tong M, Hai T, et al Efficient and rapid generation of induced pluripotent stem cells using an alternative culture medium. Cell Res. 2010;20(3):383–6. Epub 2010/02/17. 10.1038/cr.2010.26 20157334

[39] Okada M, Oka M, Yoneda Y. Effective culture conditions for the induction of pluripotent stem cells. Biochim Biophys Acta. 2010;1800(9):956–63. Epub 2010/04/27. 10.1016/j.bbagen.2010.04.004 20417254

[40] Shi Y, Do JT, Desponts C, Hahm HS, Scholer HR, Ding S. A combined chemical and genetic approach for the generation of induced pluripotent stem cells. Cell Stem Cell. 2008;2(6):525–8. Epub 2008/06/05. 10.1016/j.stem.2008.05.011 18522845

[41] Medvedev SP, Grigor'eva EV, Shevchenko AI, Malakhova AA, Dementyeva EV, Shilov AA, et al Human induced pluripotent stem cells derived from fetal neural stem cells successfully undergo directed differentiation into cartilage. Stem Cells Dev. 2011;20(6):1099–112. Epub 2010/09/18. 10.1089/scd.2010.0249 20846027

[42] Liang G, Taranova O, Xia K, Zhang Y. Butyrate promotes induced pluripotent stem cell generation. J Biol Chem. 2010;285(33):25516–21. Epub 2010/06/18. 10.1074/jbc.M110.142059 20554530 PMC2919115

[43] Mali P, Chou BK, Yen J, Ye Z, Zou J, Dowey S, et al Butyrate greatly enhances derivation of human induced pluripotent stem cells by promoting epigenetic remodeling and the expression of pluripotency-associated genes. Stem cells. 2010;28(4):713–20. Epub 2010/03/05. 10.1002/stem.402 20201064 PMC3015217

[44] Zhu S, Li W, Zhou H, Wei W, Ambasudhan R, Lin T, et al Reprogramming of human primary somatic cells by OCT4 and chemical compounds. Cell Stem Cell. 2010;7(6):651–5. Epub 2010/11/30. 10.1016/j.stem.2010.11.015 21112560 PMC3812930

[45] Zhang Z, Wu WS. Sodium butyrate promotes generation of human induced pluripotent stem cells through induction of the miR302/367 cluster. Stem Cells Dev. 2013;22(16):2268–77. Epub 2013/03/29. 10.1089/scd.2012.0650 23534850 PMC3730377

[46] Jeon BG, Coppola G, Perrault SD, Rho GJ, Betts DH, King WA. S-adenosylhomocysteine treatment of adult female fibroblasts alters X-chromosome inactivation and improves in vitro embryo development after somatic cell nuclear transfer. Reproduction. 2008;135(6):815–28. Epub 2008/02/29. 10.1530/REP-07-0442 18304987

[47] Huangfu D, Maehr R, Guo W, Eijkelenboom A, Snitow M, Chen AE, et al Induction of pluripotent stem cells by defined factors is greatly improved by small-molecule compounds. Nature biotechnology. 2008;26(7):795–7. Epub 2008/06/24. 10.1038/nbt1418 18568017 PMC6334647

[48] Huang Y, Tang X, Xie W, Zhou Y, Li D, Yao C, et al Histone deacetylase inhibitor significantly improved the cloning efficiency of porcine somatic cell nuclear transfer embryos. Cell Reprogram. 2011;13(6):513–20. Epub 2011/10/28. 22029418 10.1089/cell.2011.0032

[49] Dan J, Yang J, Liu Y, Xiao A, Liu L. Roles for Histone Acetylation in Regulation of Telomere Elongation and Two-cell State in Mouse ES Cells. Journal of cellular physiology. 2015;230(10):2337–44. 10.1002/jcp.24980 25752831 PMC4711819

[50] Dan J, Liu Y, Liu N, Chiourea M, Okuka M, Wu T, et al Rif1 maintains telomere length homeostasis of ESCs by mediating heterochromatin silencing. Dev Cell. 2014;29(1):7–19. Epub 2014/04/17. 10.1016/j.devcel.2014.03.004 24735877 PMC4720134

[51] Huang J, Wang F, Okuka M, Liu N, Ji G, Ye X, et al Association of telomere length with authentic pluripotency of ES/iPS cells. Cell Res. 2011;21(5):779–92. Epub 2011/02/02. 10.1038/cr.2011.16 21283131 PMC3203670

[52] Huang J, Deng K, Wu H, Liu Z, Chen Z, Cao S, et al Efficient production of mice from embryonic stem cells injected into four- or eight-cell embryos by piezo micromanipulation. Stem cells. 2008;26(7):1883–90. Epub 2008/05/10. 10.1634/stemcells.2008-0164 18467666

[53] Liu K, Ji G, Mao J, Liu M, Wang L, Chen C, et al Generation of porcine-induced pluripotent stem cells by using OCT4 and KLF4 porcine factors. Cell Reprogram. 2012;14(6):505–13. 23035653 10.1089/cell.2012.0047

[54] Nichols J, Smith A. Naive and primed pluripotent states. Cell Stem Cell. 2009;4(6):487–92. Epub 2009/06/06. 10.1016/j.stem.2009.05.015 19497275

[55] Hanna J, Cheng AW, Saha K, Kim J, Lengner CJ, Soldner F, et al Human embryonic stem cells with biological and epigenetic characteristics similar to those of mouse ESCs. Proc Natl Acad Sci U S A. 2010;107(20):9222–7. 10.1073/pnas.1004584107 20442331 PMC2889088

[56] Valamehr B, Robinson M, Abujarour R, Rezner B, Vranceanu F, Le T, et al Platform for induction and maintenance of transgene-free hiPSCs resembling ground state pluripotent stem cells. Stem cell reports. 2014;2(3):366–81. Epub 2014/03/29. 10.1016/j.stemcr.2014.01.014 24672758 PMC3964282

[57] Jiang J, Lv W, Ye X, Wang L, Zhang M, Yang H, et al Zscan4 promotes genomic stability during reprogramming and dramatically improves the quality of iPS cells as demonstrated by tetraploid complementation. Cell Res. 2013;23(1):92–106. Epub 2012/11/14. 10.1038/cr.2012.157 23147797 PMC3541664

[58] Chen J, Liu H, Liu J, Qi J, Wei B, Yang J, et al H3K9 methylation is a barrier during somatic cell reprogramming into iPSCs. Nature genetics. 2013;45(1):34–42. Epub 2012/12/04. 10.1038/ng.2491 23202127

[59] Stadtfeld M, Apostolou E, Ferrari F, Choi J, Walsh RM, Chen T, et al Ascorbic acid prevents loss of Dlk1-Dio3 imprinting and facilitates generation of all-iPS cell mice from terminally differentiated B cells. Nature genetics. 2012;44(4):398–405. Epub 2012/03/06. 10.1038/ng.1110 22387999 PMC3538378

[60] Muro Y, Sugiura K, Mimori T, Akiyama M. DNA mismatch repair enzymes: Genetic defects and autoimmunity. Clinica chimica acta; international journal of clinical chemistry. 2015;442C:102–9. Epub 2015/01/27. 10.1016/j.cca.2015.01.014 25619773

[61] Ji G, Liu K, Okuka M, Liu N, Liu L. Association of telomere instability with senescence of porcine cells. BMC cell biology. 2012;13:36 10.1186/1471-2121-13-36 23241441 PMC3563453

[62] Chan YS, Goke J, Ng JH, Lu X, Gonzales KA, Tan CP, et al Induction of a human pluripotent state with distinct regulatory circuitry that resembles preimplantation epiblast. Cell Stem Cell. 2013;13(6):663–75. Epub 2013/12/10. 10.1016/j.stem.2013.11.015 24315441

[63] Fang R, Liu K, Zhao Y, Li H, Zhu D, Du Y, et al Generation of naive induced pluripotent stem cells from rhesus monkey fibroblasts. Cell Stem Cell. 2014;15(4):488–96. Epub 2014/10/04. 10.1016/j.stem.2014.09.004 25280221

[64] Rodriguez A, Allegrucci C, Alberio R. Modulation of pluripotency in the porcine embryo and iPS cells. PloS one. 2012;7(11):e49079 Epub 2012/11/13. 10.1371/journal.pone.0049079 23145076 PMC3493503

[65] Petkov S, Hyttel P, Niemann H. The Small Molecule Inhibitors PD0325091 and CHIR99021 Reduce Expression of Pluripotency-Related Genes in Putative Porcine Induced Pluripotent Stem Cells. Cell Reprogram. 2014;16(4):235–40. 10.1089/cell.2014.0010 24960205

[66] Gao Y, Guo Y, Duan A, Cheng D, Zhang S, Wang H. Optimization of culture conditions for maintaining porcine induced pluripotent stem cells. DNA and cell biology. 2014;33(1):1–11. Epub 2013/11/22. 10.1089/dna.2013.2095 24256201

[67] Zhang S, Guo Y, Cui Y, Liu Y, Yu T, Wang H. Generation of intermediate porcine iPS cells under culture condition favorable for mesenchymal-to-epithelial transition. Stem cell reviews. 2015;11(1):24–38. Epub 2014/08/20. 10.1007/s12015-014-9552-x 25134796

[68] Weissbein U, Benvenisty N, Ben-David U. Quality control: Genome maintenance in pluripotent stem cells. The Journal of cell biology. 2014;204(2):153–63. 10.1083/jcb.201310135 24446481 PMC3897183

