# Supplementary material for: DNA repair and replication links to pluripotency and differentiation capacity of pig iPS cells
Source: PLoS One. 2017 Mar 2;12(3):e0173047. doi: 10.1371/journal.pone.0173047 (PMC5333863; doi:10.1371/journal.pone.0173047)
Supplement: S1 Fig — For the generation of high-quality pig iPSCs, a number of small molecules were tested. (A) Table summarizes induction factors, feeder, inducing medium, colony formation and AP staining. (B) Representative AP staining images under various conditions on day 12 of induction. (DOC) [file pone.0173047.s001.doc]

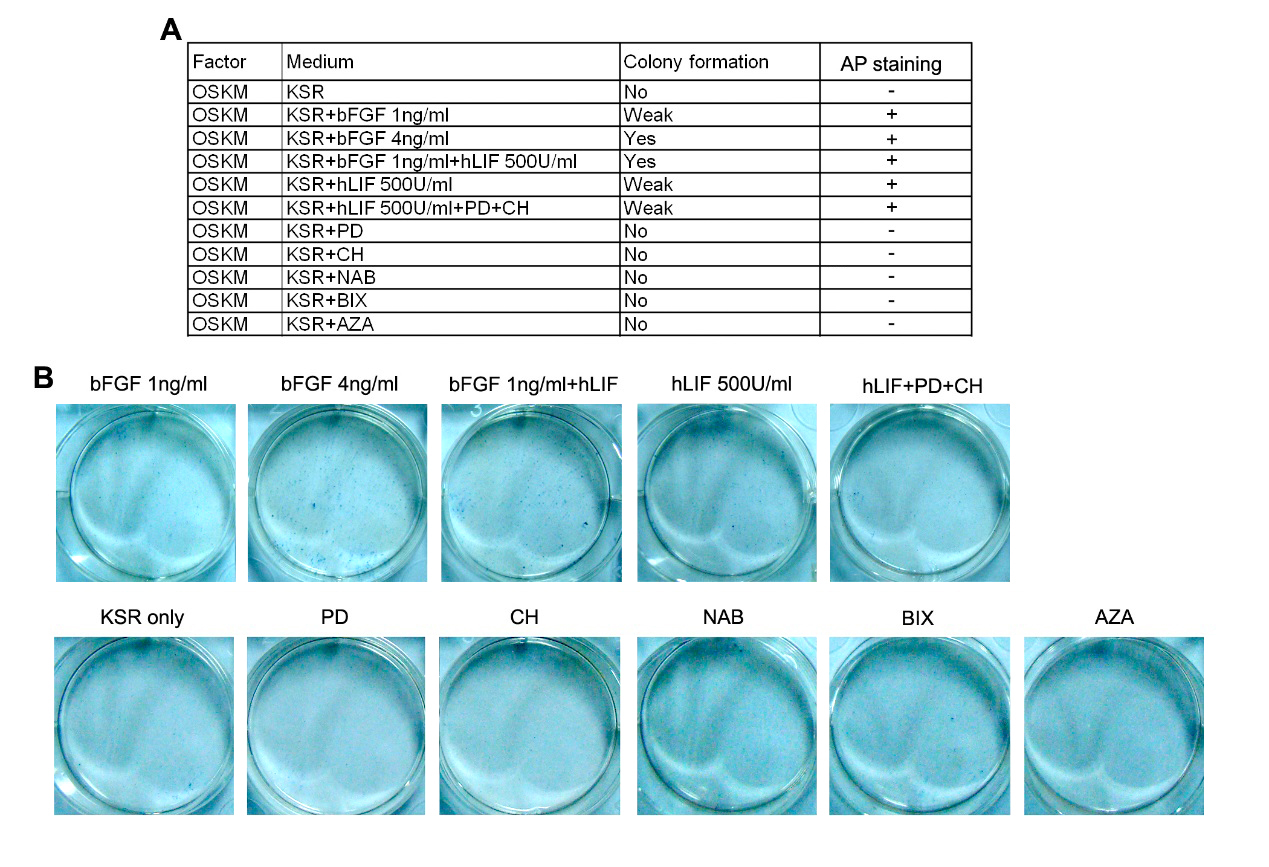


**Figure S1. bFGF and hLIF improve formation of AP positive iPSC colonies induced by OSKM.**

For the generation of high-quality pig iPSCs, a number of small molecules were tested. (A) Table summarizes induction factors, feeder, inducing medium, colony formation and AP staining. (B) Representative AP staining images under various conditions on day 12 of induction.
