# Supplementary material for: DNA repair and replication links to pluripotency and differentiation capacity of pig iPS cells
Source: PLoS One. 2017 Mar 2;12(3):e0173047. doi: 10.1371/journal.pone.0173047 (PMC5333863; doi:10.1371/journal.pone.0173047)
Supplement: S2 Fig — iPSCs induced by OSKM exhibited strong AP staining in both KSR supplemented with bFGF and mTeSR, while OKM and SKM only showed no or weak AP staining at P3. Representative AP staining images (upper) and Table summary of colony picking and passaging of iPSCs (lower) are shown. Scale bar = 100 μm. (DOC) [file pone.0173047.s002.doc]

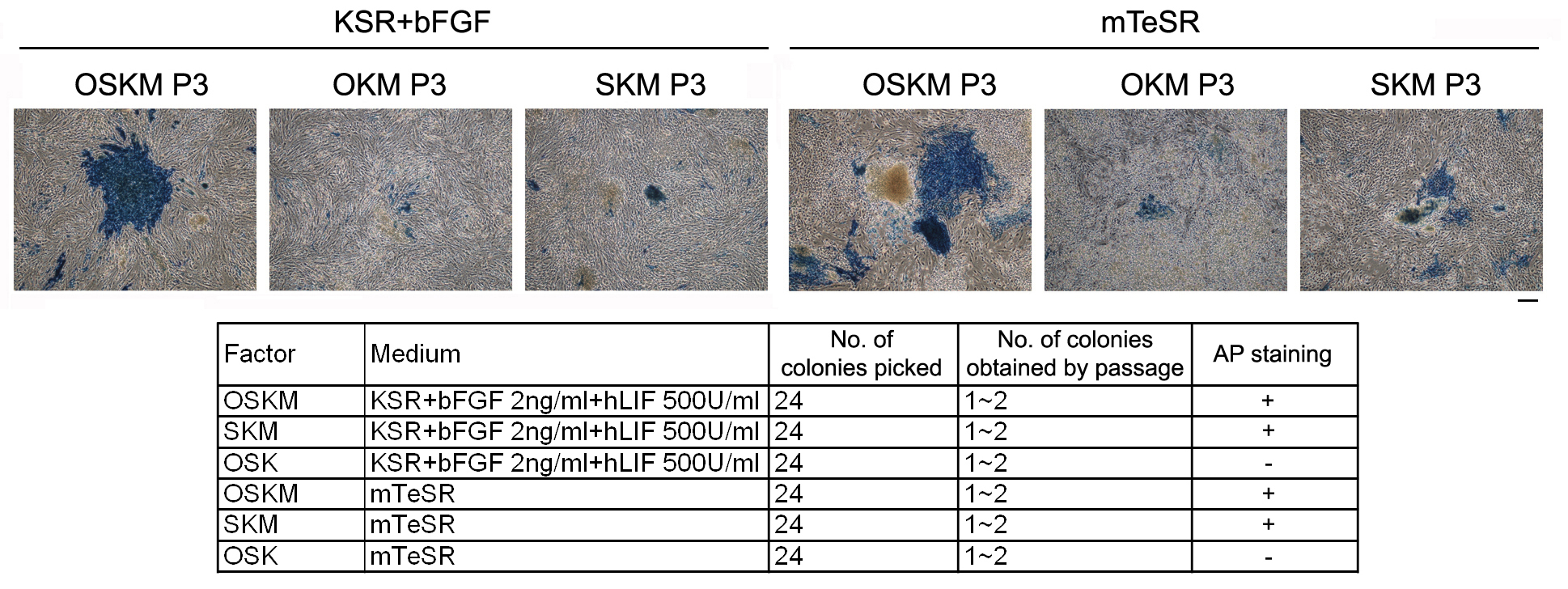


**Figure S2. Pig iPSCs induced by OSKM are maintained better relative to fewer factors.**

iPSCs induced by OSKM exhibited strong AP staining in both KSR supplemented with bFGF and mTeSR, while OKM and SKM only showed no or weak AP staining at P3. Representative AP staining images (upper) and Table summary of colony picking and passaging of iPSCs (lower) are shown. Scale bar = 100 μm.
