# Supplementary material for: DNA repair and replication links to pluripotency and differentiation capacity of pig iPS cells
Source: PLoS One. 2017 Mar 2;12(3):e0173047. doi: 10.1371/journal.pone.0173047 (PMC5333863; doi:10.1371/journal.pone.0173047)
Supplement: S3 Fig — (A) Percentage of SSEA-4 positive cells was increased when small molecules in combination of NaB, SAH and BIX01294 (named 3 chemicals) were added from day 1 to day 18 during induction. (B) Table showing methods for selection and characteristics of pig iPSCs. (C) Representative images under bright field with phase contrast optics (left) and immunofluorescence staining and microscopy (right) of pig iPSCs induced by SKM or OSKM at P3. SKM-iPSC expressed Nanog and SSEA-4 but only weak Oct4 while OSKM-iPSC expressed Nanog, Oct4 and SSEA-4. Nuclei stained with Hoechst 33342 (blue). For phase-contrast optics (Ph), Scale bar = 100 μm; for immunofluorescence images, Scale bar = 50 μm. (DOC) [file pone.0173047.s003.doc]

**
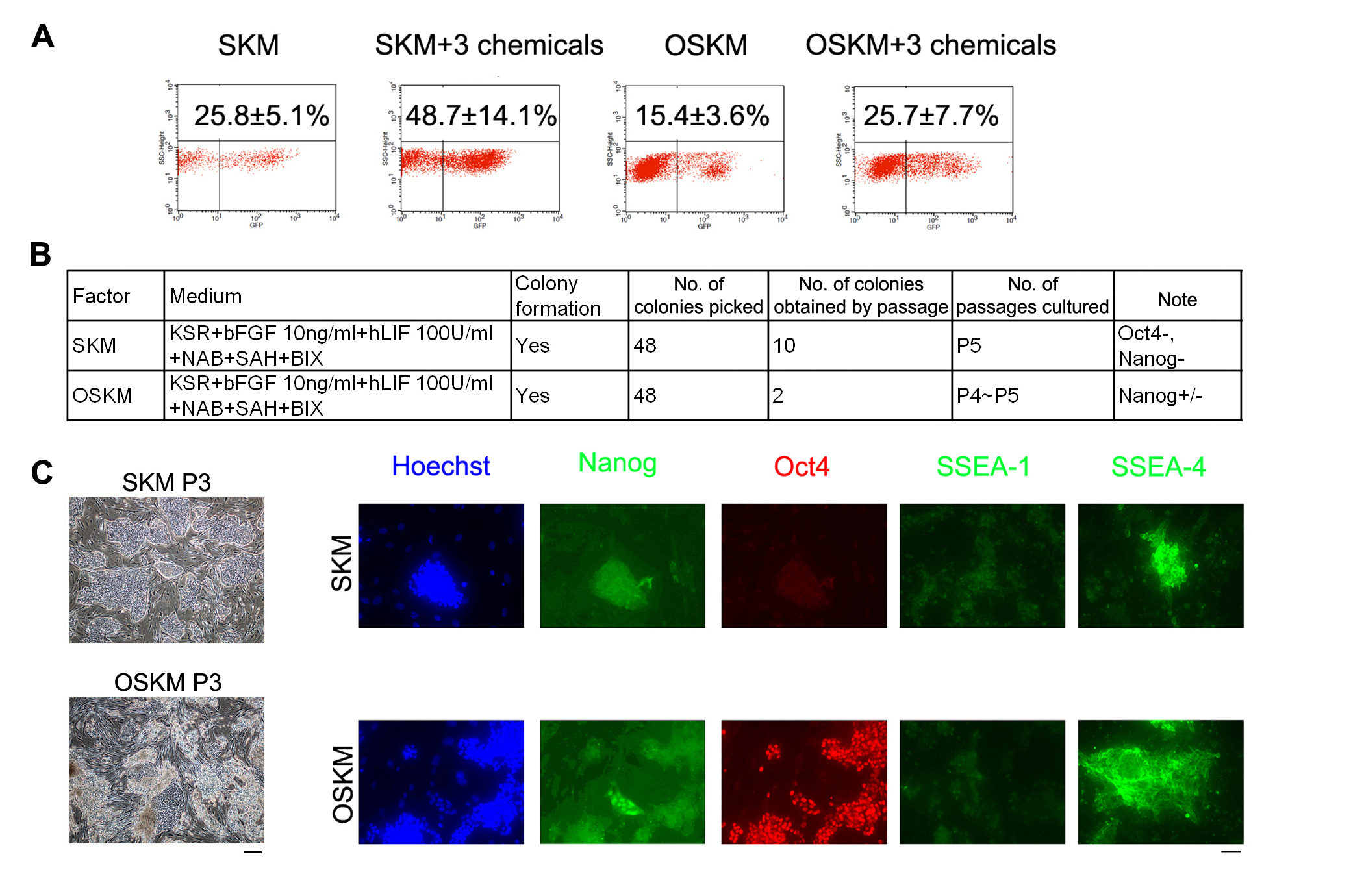
**

**Figure S3. Addition of small molecules in the establishment of pig iPSCs.**

(A) Percentage of SSEA-4 positive cells was increased when small molecules in combination of NaB, SAH and BIX01294 (named 3 chemicals) were added from day 1 to day 18 during induction. (B) Table showing methods for selection and characteristics of pig iPSCs. (C) Representative images under bright field with phase contrast optics (left) and immunofluorescence staining and microscopy (right) of pig iPSCs induced by SKM or OSKM at P3. SKM-iPSC expressed Nanog and SSEA-4 but only weak Oct4 while OSKM-iPSC expressed Nanog, Oct4 and SSEA-4. Nuclei stained with Hoechst 33342 (blue).For phase-contrast optics (Ph), Scale bar = 100 μm; for immunoﬂuorescence images, Scale bar = 50 μm.
