# Supplementary material for: DNA repair and replication links to pluripotency and differentiation capacity of pig iPS cells
Source: PLoS One. 2017 Mar 2;12(3):e0173047. doi: 10.1371/journal.pone.0173047 (PMC5333863; doi:10.1371/journal.pone.0173047)
Supplement: S4 Fig — Taihu pig embryonic fibroblast (PEF), Taihu adult pig fibroblast (PF) and Small Xiang Pig PEF were used for generation of pig iPSC by OSKM or OSKMN. Through high throughput of picking colonies, the iPSCs were derived from Small Xiang Pig PEF. (A) Table summarizing characteristics of iPSC lines (upper) and representative images under bright-field with phase contrast optics under conditions numbered 1/3/4/5/6 (lower). Scale bar = 100 μm. (B) Expression levels of endogenous Nanog were significantly higher in colonies obtained underconditions No.5 and No.6 by qPCR. Representative high quality iPSCs colonies that could be cultured for more than 5 passages were examined. (DOC) [file pone.0173047.s004.doc]

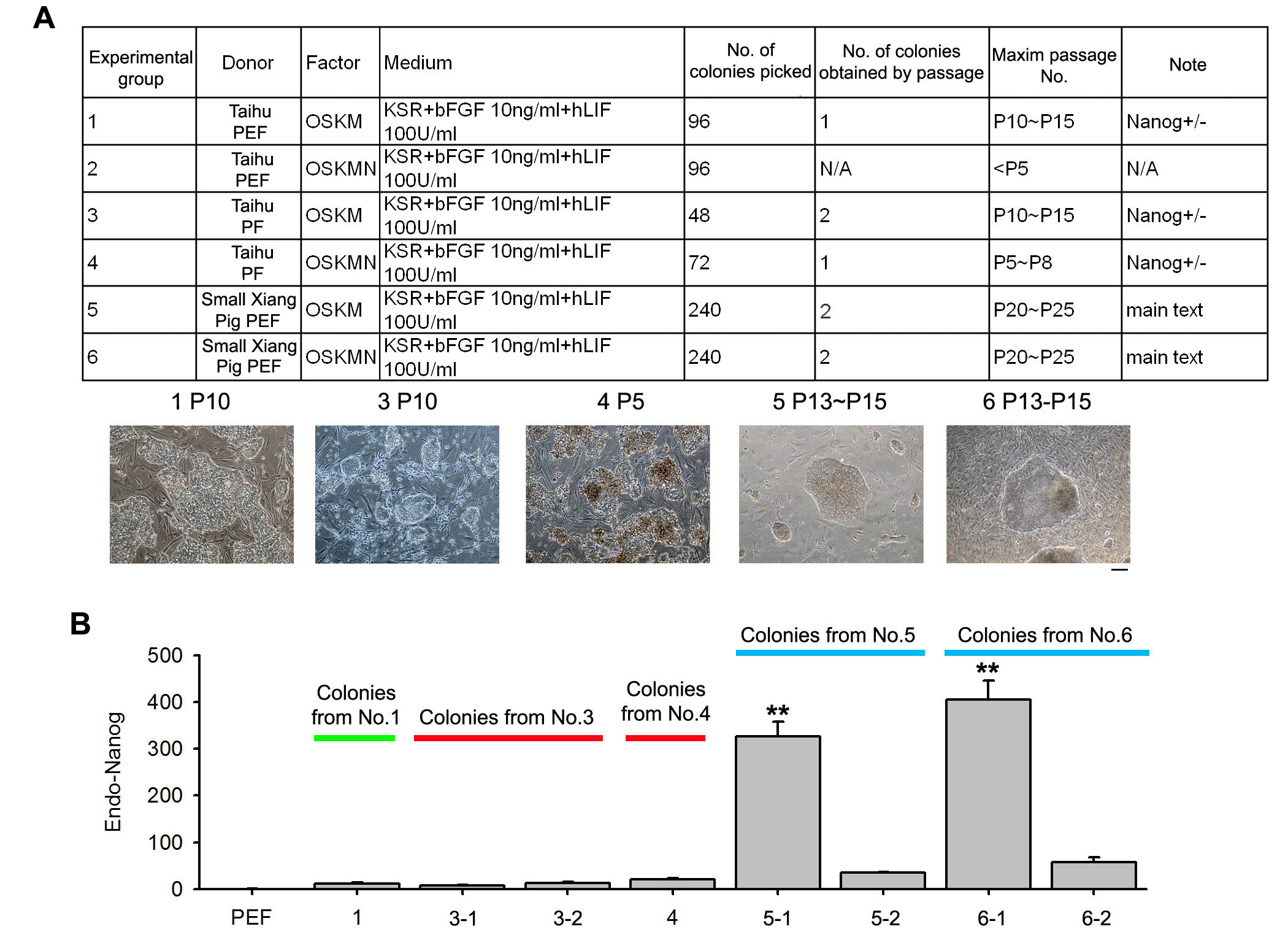


**Figure S4. High quality pig iPSCs induced by OSKM or OSKMN derived from Small Xiang Pig PEF by addition of small molecules.**

Taihu pig embryonic fibroblast (PEF), Taihu adult pig fibroblast (PF) and Small Xiang Pig PEF were used for generation of pig iPSC by OSKM or OSKMN. Through high throughput of picking colonies, the iPSCs were derived from Small Xiang Pig PEF. (A) Table summarizing characteristics of iPSC lines (upper) and representative images under bright-field with phase contrast optics under conditions numbered 1/3/4/5/6 (lower). Scale bar = 100 μm. (B) Expression levels of endogenous Nanog were significantly higher in colonies obtained underconditionsNo.5 and No.6 by qPCR. Representative high quality iPSCs colonies that could be cultured for more than 5 passages were examined.
