# Supplementary material for: DNA repair and replication links to pluripotency and differentiation capacity of pig iPS cells
Source: PLoS One. 2017 Mar 2;12(3):e0173047. doi: 10.1371/journal.pone.0173047 (PMC5333863; doi:10.1371/journal.pone.0173047)
Supplement: S5 Fig — Tgfβ is highly activated in PEF and high expression of BMP in PEF is downregulated after induction into iPSCs. Interestingly, FST for inhibition of Tgfβ signaling is upregulated in iPSCs. (DOC) [file pone.0173047.s005.doc]

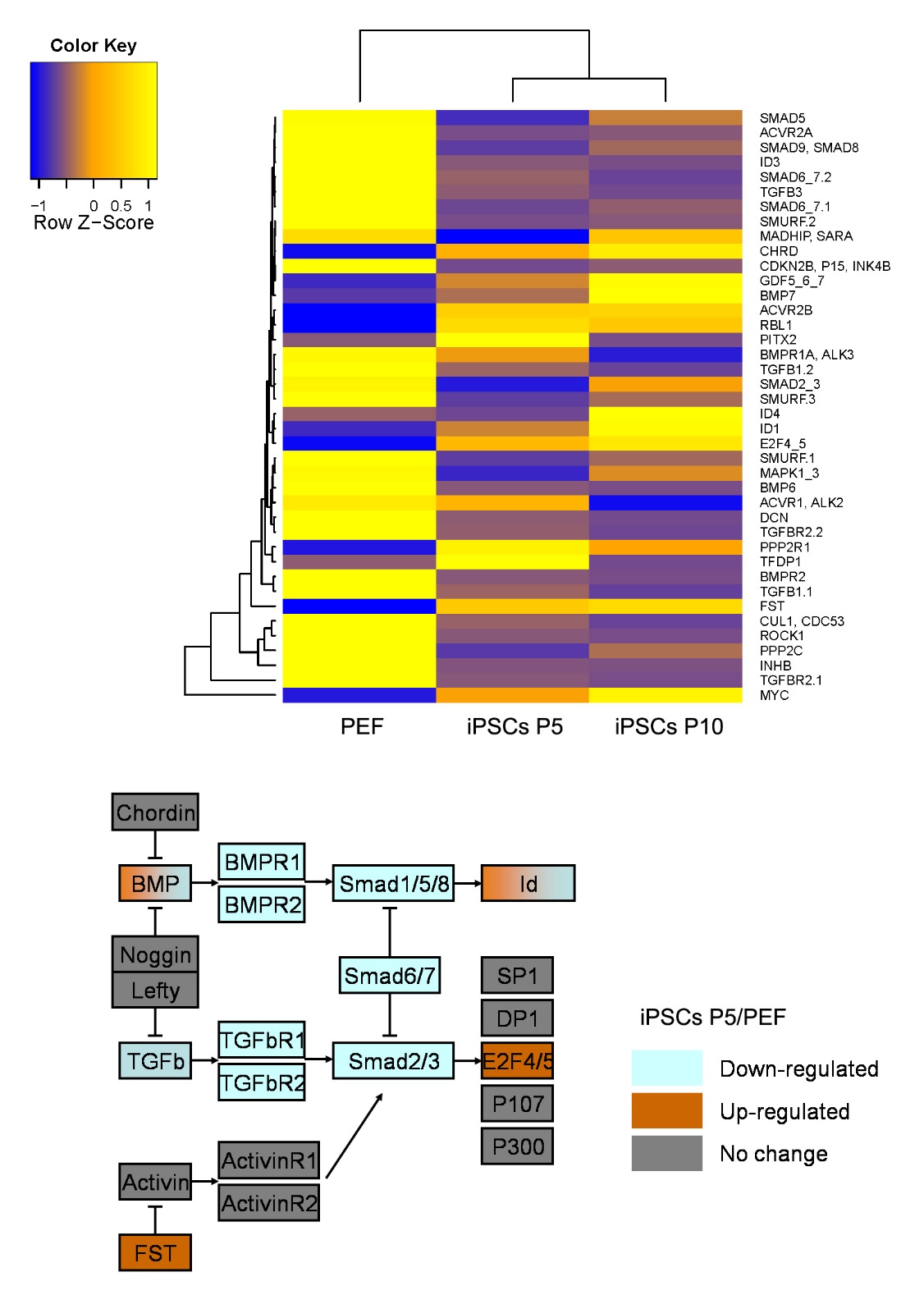


**Figure S5. TGFβ signaling pathway of pig iPSCs revealed by RNA-sequencing analysis.**

Tgfβ is highly activated in PEF and high expression of BMP in PEF is downregulated after induction into iPSCs. Interestingly, FST for inhibition of Tgfβ signaling is upregulated in iPSCs.
