# Supplementary material for: DNA repair and replication links to pluripotency and differentiation capacity of pig iPS cells
Source: PLoS One. 2017 Mar 2;12(3):e0173047. doi: 10.1371/journal.pone.0173047 (PMC5333863; doi:10.1371/journal.pone.0173047)
Supplement: S6 Fig — Wnt8, Wnt11.1, Wnt11.2, Tcf, and Ruvbl (Ino80) of Wnt signaling is upregulated in iPSCs at P5, but downregulated in iPSCs at P10. (DOC) [file pone.0173047.s006.doc]

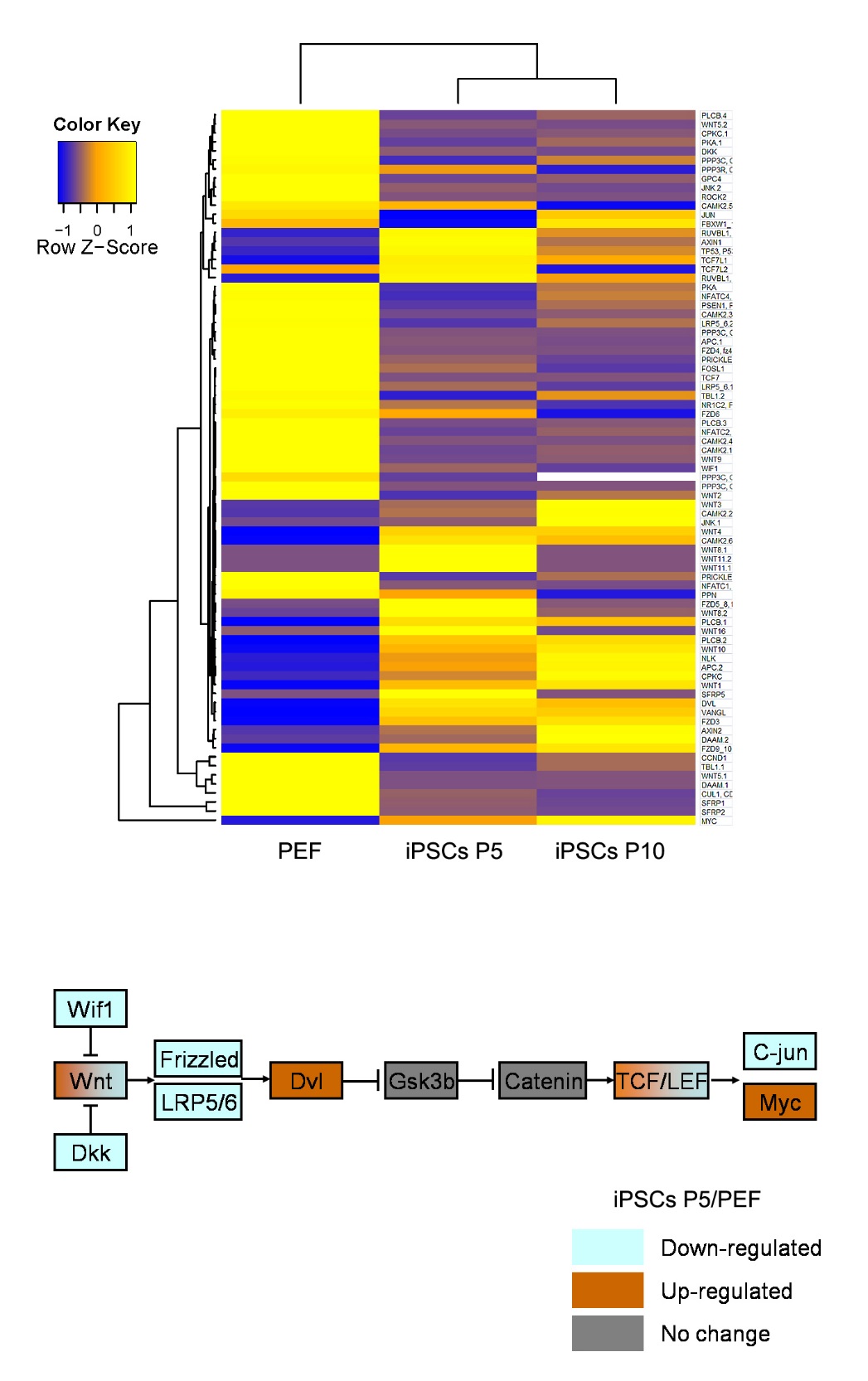


**Figure S6. WNT signaling pathway of pig iPSCs revealed by RNA-sequencing.**

Wnt8, Wnt11.1, Wnt11.2, Tcf, and Ruvbl (Ino80) of Wnt signaling is upregulated in iPSCs at P5, but downregulated in iPSCs at P10.
