# Supplementary material for: DNA repair and replication links to pluripotency and differentiation capacity of pig iPS cells
Source: PLoS One. 2017 Mar 2;12(3):e0173047. doi: 10.1371/journal.pone.0173047 (PMC5333863; doi:10.1371/journal.pone.0173047)
Supplement: S7 Fig — EFG, EGFR, PDGF, JNK of MAPK pathway are highly expressed in PEF, but downregulated in early and late passages of iPSCs in general. (DOC) [file pone.0173047.s007.doc]

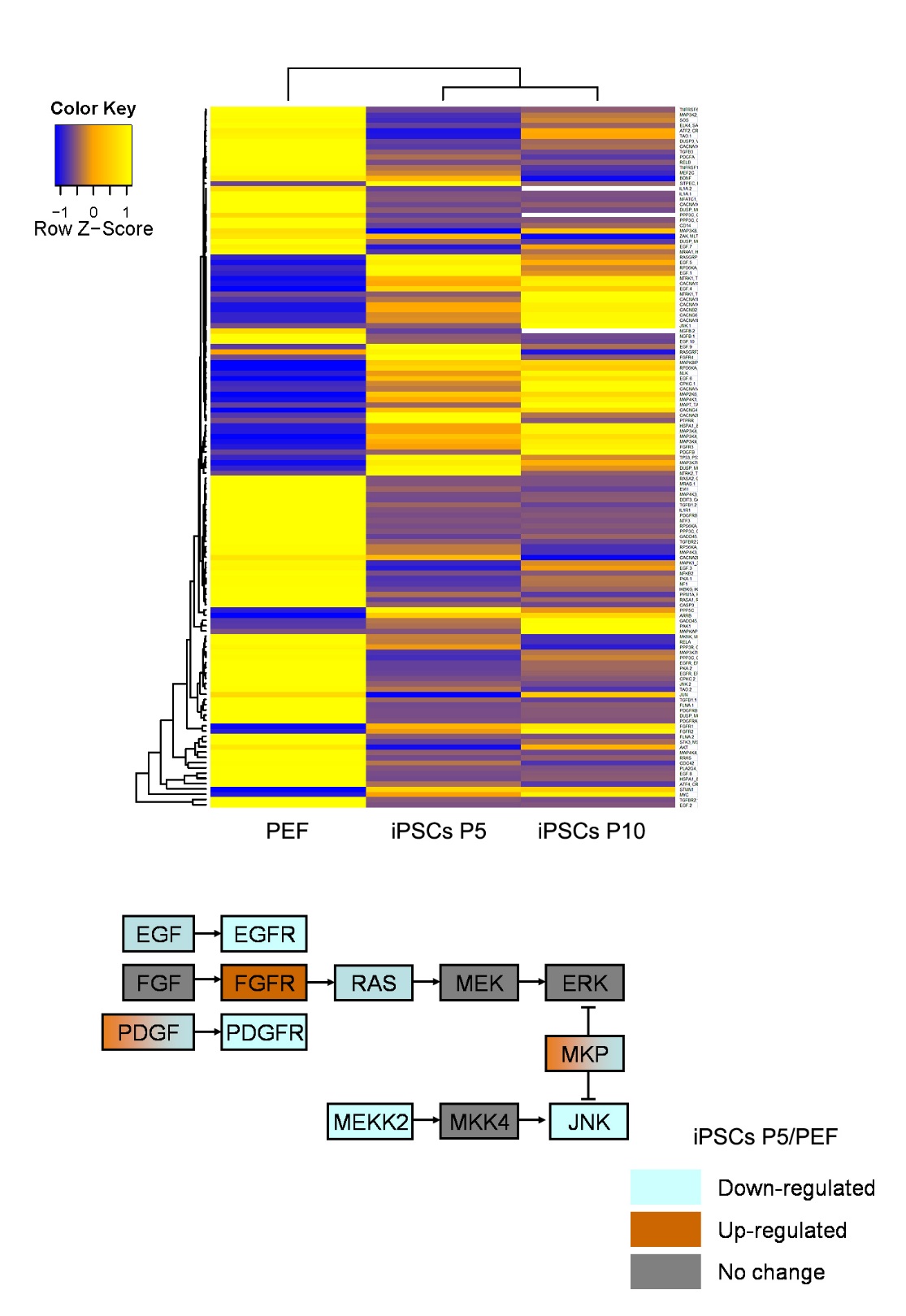


**Figure S7. MAPK signaling pathway revealed by RNA-sequencing.**

EFG, EGFR, PDGF, JNK of MAPK pathway are highly expressed in PEF, but downregulated in early and late passages of iPSCs in general.
