# Supplementary material for: DNA repair and replication links to pluripotency and differentiation capacity of pig iPS cells
Source: PLoS One. 2017 Mar 2;12(3):e0173047. doi: 10.1371/journal.pone.0173047 (PMC5333863; doi:10.1371/journal.pone.0173047)
Supplement: S8 Fig — Signaling associated with apoptosis such as Casp3/8/9 is greatly reduced in iPSCs at P5 and P10. Some pro-apoptosis genes (p53) is upregulated, while anti-apoptosis genes (PIK3) also upregulated in iPSCs. (DOC) [file pone.0173047.s008.doc]

**
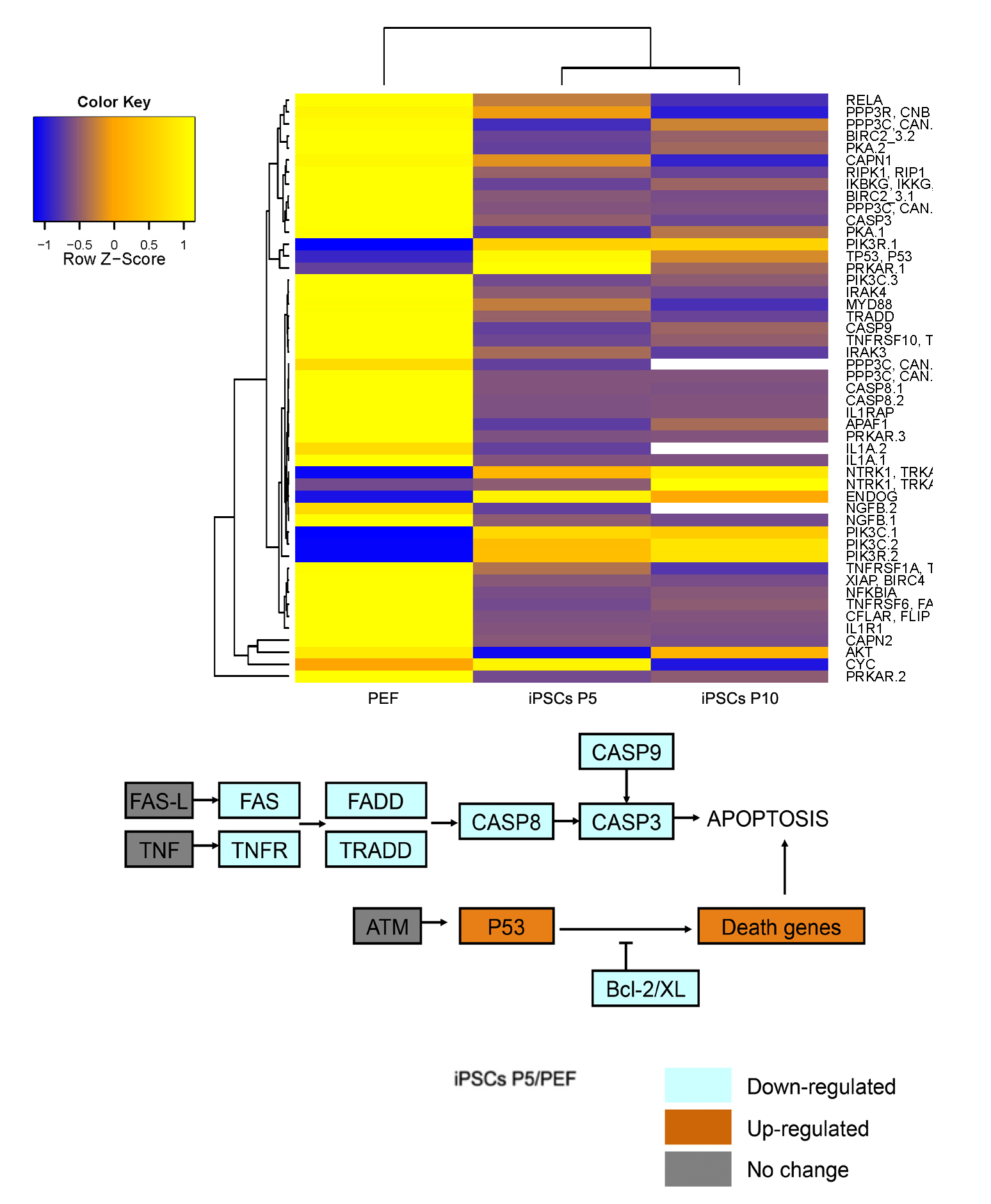
**

**Figure** **S8. Apoptosis signaling pathway analysis by RNA-sequencing.**

Signaling associated with apoptosis such as Casp3/8/9 is greatly reduced in iPSCs at P5 and P10. Some pro-apoptosis genes (p53) is upregulated, while anti-apoptosis genes (PIK3) also upregulated in iPSCs.
