# Supplementary material for: DNA repair and replication links to pluripotency and differentiation capacity of pig iPS cells
Source: PLoS One. 2017 Mar 2;12(3):e0173047. doi: 10.1371/journal.pone.0173047 (PMC5333863; doi:10.1371/journal.pone.0173047)
Supplement: S9 Fig — Signaling associated with Nucleotide excision repair (NER) such as Pold1 and Pold3 is increased in iPSCs at P5 and P10. (DOC) [file pone.0173047.s009.doc]

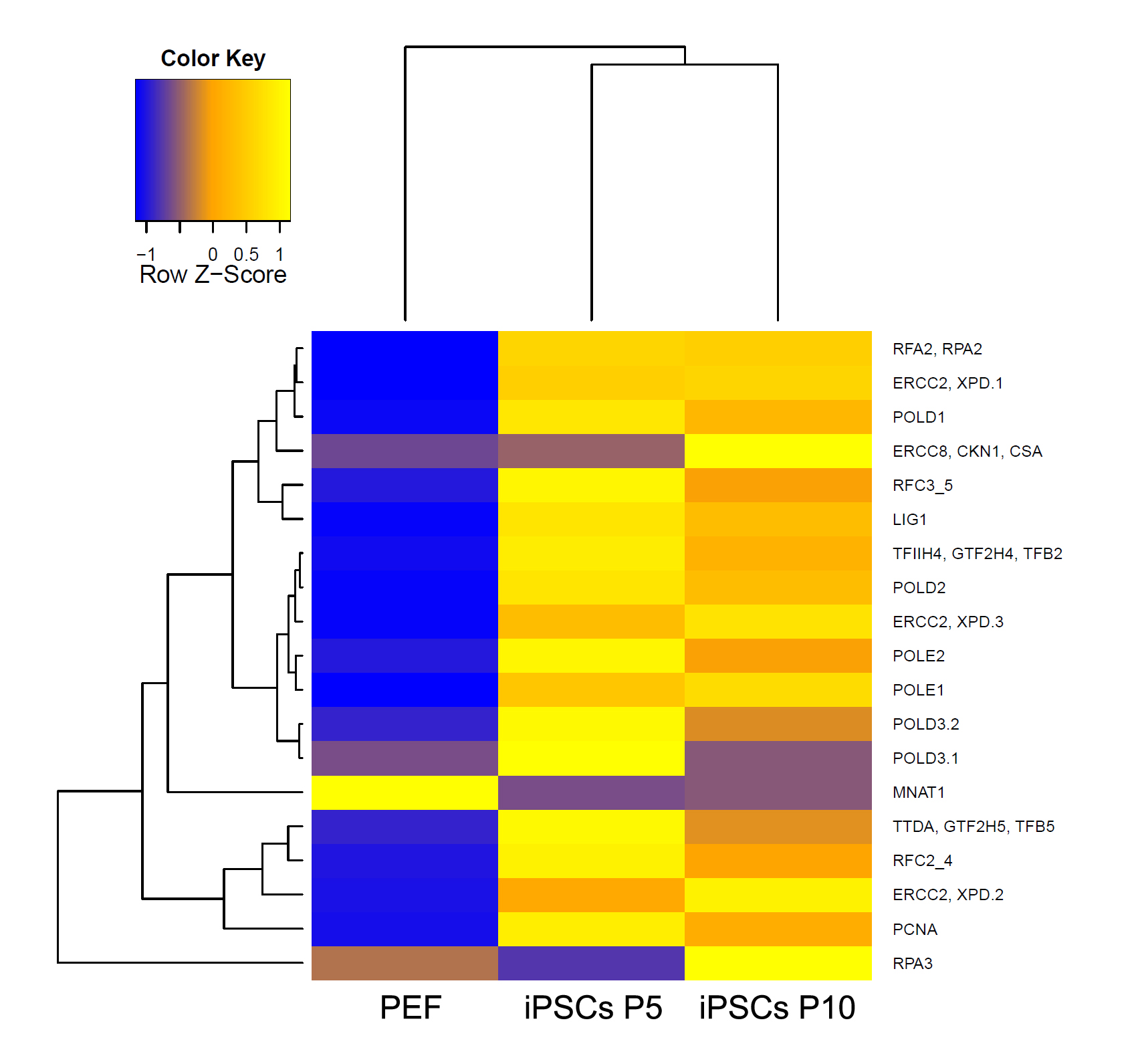


**Figure S9. Nucleotide excision repair analysis by RNA-sequencing.**

Signaling associated with Nucleotide excision repair (NER) such as Pold1 and Pold3 is increased in iPSCs at P5 and P10.
