# Supplementary material for: DNA repair and replication links to pluripotency and differentiation capacity of pig iPS cells
Source: PLoS One. 2017 Mar 2;12(3):e0173047. doi: 10.1371/journal.pone.0173047 (PMC5333863; doi:10.1371/journal.pone.0173047)
Supplement: S1 Table — (DOC) [file pone.0173047.s010.doc]

**Table S1. Characteristics of pig iPSCs produced by different laboratories.**
