# Supplementary material for: DNA repair and replication links to pluripotency and differentiation capacity of pig iPS cells
Source: PLoS One. 2017 Mar 2;12(3):e0173047. doi: 10.1371/journal.pone.0173047 (PMC5333863; doi:10.1371/journal.pone.0173047)
Supplement: S2 Table — (DOC) [file pone.0173047.s011.doc]

**Table S2. Primers used for qPCR analysis.**

| Gene name | Primer | Genebank accession number |
| --- | --- | --- |
| endo-Oct4 | F: CTTCACCACCCTGTACTCCTC | **ENSSSCT00000001516** |
|  | R: GCTTCTCTCCCTAGCTCACC |  |
| endo-Sox2 | F: CAGACTTCACATGTCCCAGCACTA | **ENSSSCT00000012883** |
|  | R: CTTACTCTCCTCCCATTTCCCTCT |  |
| endo-Klf4 | F: GAGGGAAGACCAGAATCCCTTGTA | **ENSSSCT00000005981** |
|  | R: TAGAACCAAGACTCACCAAGCACC |  |
| endo-cMyc | F: CACAGCTTACATCCTGTCTGTCCA | **ENSSSCT00000006548** |
|  | R: GCCATTCTAGTTCCTCCCTCCAAT |  |
| endo-Nanog | F: CCTACAATCCAGCTCTTTGG | NM_001129971 |
|  | R: CTCAGGCATTGGTGAAGATT |  |
| ex-Oct4 | F: GACGGCATCGCAGCAGCTTGGATACAC | * |
|  | R: GAGAAGGCGAAGTCGGAAG |  |
| ex-Sox2 | F: GACGGCATCGCAGCAGCTTGGATACAC | * |
|  | R: GGCTGTTCTTCTGGTTGC |  |
| ex-Klf4 | F: GACGGCATCGCAGCAGCTTGGATACAC | * |
|  | R: GTCTTTGCTTCATGTGGG |  |
| ex-cMyc | F: GACGGCATCGCAGCAGCTTGGATACAC | * |
|  | R: GTTGGTGAAGCTGACGTTG |  |
| ex-Nanog | F: GGTACGGGAATTCAAGCTTAT | * |
|  | R: CATCTGCAAGGAGGCATAAT |  |
| Rex1 | F: CTCTCTCAACAGGTGCTTAC | **ENSSSCT00000007665** |
|  | R: AAAGTCAGTGCTGGGTATTT |  |
| Sall4 | F: ATCCACCTCCGCTCCCATACC | NM_001114673 |
|  | R: CGTTGCCTGCCGTCATCTTGT |  |
| E-cadherin | F: GAACCCACAGCCTCATGTCA | NM_001163060 |
|  | R: CGGTCGTTGAACTCGATGGT |  |
| Lin28 | F: TCTTTGTGCACCAGAGTAAGC | NM_001123133 |
|  | R: ATTCCAAGCCCTTAGCAGAC |  |
| Dppa2 | F: GATACAGAAGGTTGGGTTCG | XM_003358822 |
|  | R: TAGCACATTCAGGGCATAAC |  |
| BMP4 | F: CTCGCTCTATGTGGACTTCAG | NM_001101031 |
|  | R: GATGGCATGATTGGTTGAGTTG |  |
| Tert | F: GAAAGCCAGAAACGCAGGGAT | NM_001244300 |
|  | R: CCAGAAGACAGCTGTAGGTAACG |  |
| Terc | F: GTCTAACCCTAACTGAAAGAGGCG | AF221920 |
|  | R: TGTTTCAGACTGGATGGTGGATGG |  |
| p53 | F: AGTGGCAACTTGCTGGGACG | NM_213824 |
|  | R: TGGTGGGCAGTGCTCGCTTA |  |
| β-actin | F: TGCGGCATCCACGAAACTAC | DQ845171 |
|  | R: TTCTGCATCCTGTCGGCGAT |  |

*Primers for exogenous transcription factor were based on the plasmid.
